# B-Cell Epitopes in GroEL of *Francisella tularensis*

**DOI:** 10.1371/journal.pone.0099847

**Published:** 2014-06-26

**Authors:** Zhaohua Lu, Michael J. Rynkiewicz, Guillermo Madico, Sheng Li, Chiou-Ying Yang, Hillary M. Perkins, Seshi R. Sompuram, Vani Kodela, Tong Liu, Timothy Morris, Daphne Wang, Marly I. Roche, Barbara A. Seaton, Jacqueline Sharon

**Affiliations:** 1 Department of Pathology and Laboratory Medicine, Boston University School of Medicine, Boston, Massachusetts, United States of America; 2 Department of Physiology and Biophysics, Boston University School of Medicine, Boston, Massachusetts, United States of America; 3 Department of Medicine, Boston University School of Medicine, Boston, Massachusetts, United States of America; 4 Department of Medicine, University of California, San Diego, School of Medicine, San Diego, California, United States of America; 5 Institute of Molecular Biology, National Chung Hsing University, Taichung, Taiwan; 6 Medical Discovery Partners, LLC, Boston, Massachusetts, United States of America; Wadsworth Center, New York State Dept. Health, United States of America

## Abstract

The chaperonin protein GroEL, also known as heat shock protein 60 (Hsp60), is a prominent antigen in the human and mouse antibody response to the facultative intracellular bacterium *Francisella tularensis* (Ft), the causative agent of tularemia. In addition to its presumed cytoplasmic location, FtGroEL has been reported to be a potential component of the bacterial surface and to be released from the bacteria. In the current study, 13 IgG2a and one IgG3 mouse monoclonal antibodies (mAbs) specific for FtGroEL were classified into eleven unique groups based on shared VH-VL germline genes, and seven crossblocking profiles revealing at least three non-overlapping epitope areas in competition ELISA. In a mouse model of respiratory tularemia with the highly pathogenic Ft type A strain SchuS4, the Ab64 and N200 IgG2a mAbs, which block each other’s binding to and are sensitive to the same two point mutations in FtGroEL, reduced bacterial burden indicating that they target protective GroEL B-cell epitopes. The Ab64 and N200 epitopes, as well as those of three other mAbs with different crossblocking profiles, Ab53, N3, and N30, were mapped by hydrogen/deuterium exchange–mass spectrometry (DXMS) and visualized on a homology model of FtGroEL. This model was further supported by its experimentally-validated computational docking to the X-ray crystal structures of Ab64 and Ab53 Fabs. The structural analysis and DXMS profiles of the Ab64 and N200 mAbs suggest that their protective effects may be due to induction or stabilization of a conformational change in FtGroEL.

## Introduction

*Francisella tularensis* (Ft), the Gram negative facultative intracellular bacterium that causes tularemia, has been classified by the Centers for Disease Control and Prevention as a category A Tier 1 priority pathogen, a likely bioterrorism agent [1]–[4]. As few as 10 bacteria can cause respiratory tularemia, the most severe form of the disease, with up to 30% mortality if untreated [1]–[5]. Even when treated with antibiotics, respiratory tularemia is still associated with considerable morbidity and up to 2% mortality [2]–[6]. An attenuated type B live vaccine strain (LVS) partially protects against the highly virulent type A Ft in humans but is not currently licensed due to safety concerns [6], [7]. Understanding the mechanisms of anti-Ft immune protection and identification of protective Ft antigens and epitopes will facilitate the development of potentially safer, subunit Ft vaccines.

Although T cell immunity is essential for protection against Ft [8]–[12], B cells are required for anti-Ft memory [13], and polyclonal IgG antibodies to Ft can transfer resistance against the bacteria to naïve hosts, including humans [14]–[23]. The best known target of protective Ft antibodies is the *O*-polysaccharide (O-antigen), which comprises the Ft capsule [24], [25] and is a component of the Ft lipopolysaccharide (LPS) [17], [22], [26]–[34]. We and others have described O-antigen mAbs that confer or prolong survival in mice infected subcutaneously, intranasally or intradermally with LVS or the highly virulent Ft type A strain SchuS4 [29], [31]–[33]. Passive administration of mAbs specific for Ft outer membrane proteins FopA or LpnA was also reported to prolong survival of mice infected intradermally with lethal doses of LVS [33] and passive transfer of FopA-immune sera was shown to protect mice against lethal intradermal LVS challenge [35].

The current study adds GroEL to this limited list of Ft targets of protective antibodies. GroEL is a molecular chaperone found in many bacteria, whose expression is upregulated during stress conditions, such as elevated temperatures or high concentration of oxygen radicals [36]–[38]. It is therefore also known as chaperone protein 60 (Cpn60) and heat shock protein 60 (Hsp60) [39], [40]. Hsp60 proteins are also found in eukaryotic organisms including mammals, with over 55% identity between bacterial and mammalian versions [41]. Hsp60s belong to the family of type I chaperonins, 14-mer homopolymers with two stacked heptamer rings which form a hydrophobic cavity that binds to improperly-folded proteins. Each monomer consists of 19 β-strands (1–19) and 18 α-helices (A–R) [42] that form an equatorial domain (close to the junction of the two rings) and an apical domain (farthest from the junction of the two rings), connected by an intermediate domain [36], [37], [43]. ATP-binding to the equatorial domains of one of the heptamer rings of GroEL initiates conformational rearrangement of the monomers in the affected heptamer ring. The first rearrangement opens up the central cavity, which allows entry and binding of unfolded protein substrate in the central cavity, and binding of the cofactor Cpn10 (designated GroES in bacteria) to the apical domains of the heptamer ring, encapsulating the improperly folded protein. Additional conformational changes, induced by GroES binding, result in switching of the exposed central-cavity surface of the apical domains from hydrophobic to polar by elevation and rotation of the apical domains [42], to allow refolding of the improperly folded protein substrate. The time course of refolding is dictated by the hydrolysis of ATP to ADP [44], [45]. After hydrolysis, ATP and substrate binding to the other heptamer ring causes release of ADP, GroES, and the protein substrate [36], [37], [43].

In addition to their intra-cytoplasmic or intra-mitochondrial location, both bacterial and mammalian Hsp60s have been shown to be expressed on the cell membrane and to be secreted or released from the Hsp60-producing cell [46]–[51]. Furthermore, Hsp60s have been implicated in stimulation of both innate responses, by interacting with TLR2 or TLR4 either directly or via other molecules that attach to their hydrophobic regions such as LPS [52], and in B and T cell adaptive responses [53].

GroEL is an immunodominant antigen in many bacterial infections, inducing both B and T cell responses [51], [53]–[66]. Depending on the pathogen, these responses have been shown to be protective [56], [57], [60], [62], [63], [66], non-protective [55], [57], [58], [61], or pathogenic by leading to formation of excessive amounts of immune complexes and resultant inflammation [67] or to autoimmunity due to crossreaction with host Hsp60 [41], [67]. However, immunization with bacterial GroEL or with peptides that crossreact with host Hsp60 has been shown to protect against or ameliorate autoimmune disease through induction of regulatory T cells [68]–[70].

In the case of Ft, GroEL was identified as a potentially surface-exposed LVS protein by mass spectrometry [50], [51] and was found in culture filtrates of LVS and of a fresh clinical isolate [49]. It was reported to synergize with Ft LPS to induce secretion of the CXCL8 chemokine by human monocyte-derived macrophages [71]. FtGroEL was identified as an immunoreactive protein both in sera from mice immunized with LVS [54], [72], [73] and in sera from tularemia patients [51], [74]. It was also reported to stimulate IFN-γ-secreting CD4^+^ and CD8^+^ T cells in LVS-infected mice [49] and to be a target of human αβ T cells [75] and of Ft-specific T cell hybridomas [76]. However, host protection by FtGroEL antibodies has not been demonstrated, and one study concluded that the protection of mice against Ft type B strains by intradermal immunization with FtGroEL may be due, at least in part, to co-purified LPS [77].

In one of our previous studies on mouse Ft mAbs, we generated an IgG3 GroEL mAb, Ab12, which showed trending towards prolongation of survival in mice infected intranasally with LVS [32]. In the current study, we generated and characterized 13 IgG2a mAbs specific for FtGroEL. We show that two of these mAbs, Ab64 and N200, which block each other’s binding to FtGroEL in competition ELISA and are sensitive to the same two FtGroEL mutations, reduce bacterial burden in mice infected intranasally with SchuS4. And we map their partially overlapping target epitopes and those of three mAbs with other crossblocking profiles (Ab53, N3 and N30) by hydrogen/deuterium exchange - mass spectrometry (DXMS) and visualization on a homology model of FtGroEL. This model is supported by the computational docking of the X-ray crystal structures of Ab64 and Ab53 Fabs, guided by the DXMS and competition ELISA data.

## Results and Discussion

### Binding Properties of the First Three FtGroEL mAbs

The first FtGroEL mAb we generated, Ab12 (IgG3,κ), was derived from a mouse immunized with live LVS and its target antigen was identified by proteome microarray analysis [32]. Because antibodies of the IgG2a isotype, the mouse analog of human IgG1 [78], have been associated with immune protection in mouse models of tularemia [32], [79], we sought to obtain FtGroEL IgG2a mAbs. To that end, we generated hybridomas from mice immunized with various regimens of live LVS and GroEL-enriched killed Ft preparations adjuvanted with CpG-containing oligodeoxynucleotide, which favors Th1 responses with production of IgG2a in mice and IgG1 in humans [80], [81].

**Figure 1:**
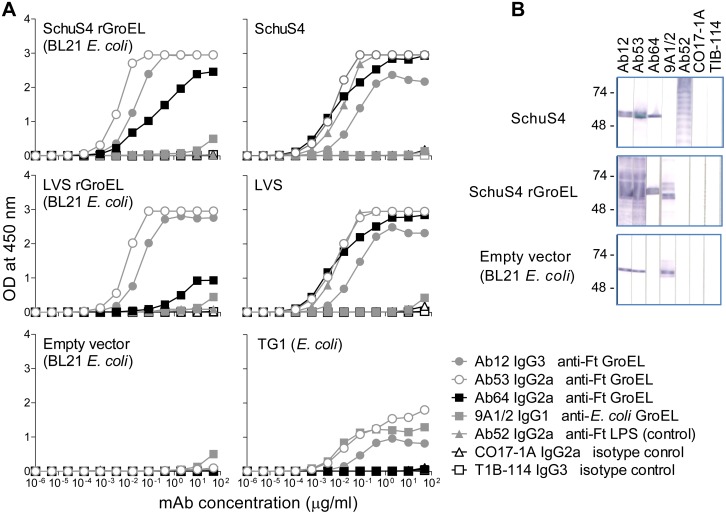
Ab12, Ab53, and Ab64 bind FtGroEL but only Ab12 and Ab53 crossreact with *E. coli*. (**A**) ELISA (data from one of two experiments with similar results are shown). Lysates of *E. coli* BL21 transformed with pET14b vector containing Ft SchuS4 GroEL DNA, Ft LVS GroEL DNA, or no insert (empty vector) were coated on ELISA plates in the left panels, and greater than 10-fold higher concentrations of SchuS4, LVS, or *E. coli* TG1 lysates were coated on ELISA plates in the right panels. The coated plates were probed with serial dilutions of the indicated mAbs. (**B**) Western blot. Equivalent concentrations of lysates (based on OD_600_ of the bacterial cultures) of SchuS4 or of BL21 transformed with SchuS4 rGroEL-vector or empty vector were electrophoresed in preparative 4–15% polyacrylamide gels under denaturing conditions and, after transfer to nitrocellulose, strips were probed with 10 µg/ml of the indicated mAbs. The positions of prestained molecular weight standards (in kDa) are indicated.

The first two of the new mAbs to be characterized, Ab53 and Ab64, both IgG2a,κ, were further tested for ELISA and Western blot reactivity to a lysate of *E. coli* BL21 expressing SchuS4 or LVS recombinant (r) GroEL. As shown in Figure 1A left panels, Ab53 and Ab64, like Ab12, bound to lysates of both SchuS4 rGroEL- and LVS rGroEL-expressing *E. coli*, in ELISA, but not to *E. coli* that had been transformed with empty vector, demonstrating specificity for FtGroEL. The reactivity of all three mAbs with both LVS and SchuS4 GroEL was expected because the two proteins differ only at three amino acids (out of 544 amino acids, NCBI Protein Blast http://blast.ncbi.nlm.nih.gov/Blast.cgi).

Ab64 showed lower binding potency than Ab53, the other IgG2a anti-GroEL mAb, especially on LVS rGroEL. The binding potency of Ab12 cannot be directly compared to that of Ab64 and Ab53 because it is of a different isotype (IgG3), and ELISA results are affected by the ability of the secondary antibodies to detect different primary antibody isotypes. Ab64 also showed lower binding potency than Ab53 on SchuS4 and LVS lysates (Figure 1A right panels), although the difference was not as pronounced as on the lysates of bacteria expressing rGroEL, which were coated at more than 10-fold lower concentrations due to the over-expressed protein. The lower binding of Ab64 to rGroEL, and especially to LVS rGroEL, may reflect imperfect folding of the recombinant proteins, which may affect the binding of Ab64 but much less, if at all, of Ab53.

Crossreactivity of Ab12 and Ab53, but not of Ab64, with *E. coli* (strain TG1) was detected with the higher coating concentration of bacterial lysates (Figure 1A bottom right panel), although equivalent binding to that with SchuS4 and LVS lysates, assessed in the linear parts of all curves (at OD 0.8), required 13.1–13.5 and 15.3–17.6-fold higher mAb concentration for Ab53 and Ab12, respectively (Figure 1A right panels). As expected, the IgG1 *E. coli* (Ec) GroEL mAb 9A1/2, used as positive control, also bound to TG1 lysate (Figure 1A bottom right panel) although the binding cannot be compared directly to that of Ab53 and Ab64 because of the different isotypes of the three mAbs. The crossreactivity of Ab53 and Ab12, but not of Ab64, with EcGroEL was also seen on Western blots of both lysate of BL21 expressing SchuS4 rGroEL and lysate of BL21 that had been transformed with empty vector, where the EcGroEL monomer can be distinguished from the FtGroEL-His tag monomer because of the lower apparent molecular weight of the former (Figure 1B).

**Figure 2:**
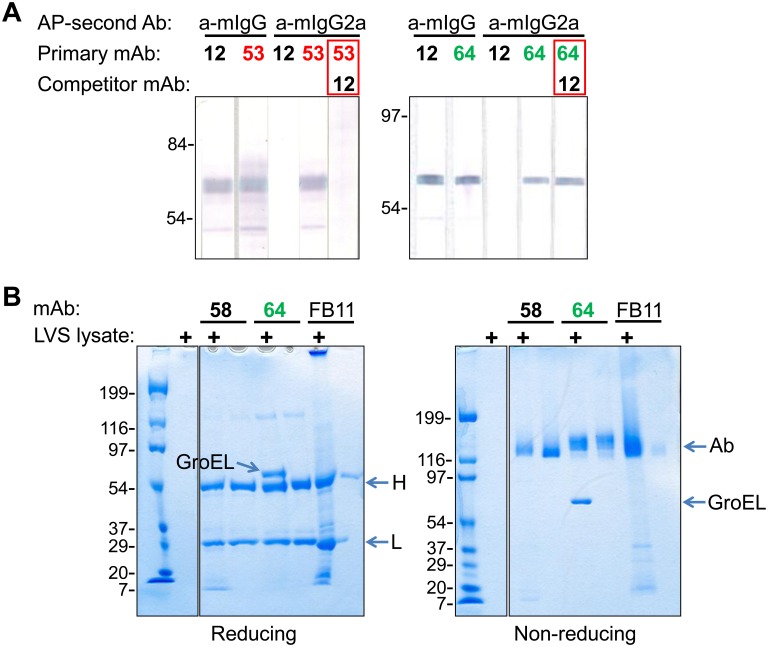
Ab64 binds to a different FtGroEL epitope than Ab12 and Ab53. (**A**) Isotype-specific competition WB. LVS lysate nitrocellulose strips were pretreated with either 800 µg/ml of Ab12 (competitor mAb, when indicated), or were mock-treated, and then 5 µg/ml of Ab12, Ab53 or Ab64 (primary mAb) was incubated with the membrane strips. Anti (a)-m (mouse)-IgG (H+L, binds IgG1, IgG2b and IgG3) or a-m-IgG2a-specific alkaline phosphatase (AP) conjugates (second Ab) were used for development, as indicated. (**B**) Immunoprecipitation. Twelve µg of the indicated mAb were incubated with 100 µl (0.25 OD_600_) of LVS lysate (+) or just buffer, followed by capture of the antibody: antigen complex with 7.5 µl of Protein G agarose resin. After washing the resin to remove unbound components of the sample, Ab and Ab-bound antigen were visualized by SDS-PAGE on 4–15% polyacrylamide gradient gels under reducing (and boiling) or non-reducing conditions. The positions of prestained molecular weight standards (in kDa) are indicated. The positions of Ab, Ab H and L chains, and GroEL are indicated by arrows. Ft mAbs Ab58 (IgG1, anti-histone-like protein HU form B) and FB11 (IgG2a, anti-LPS) were used as specificity controls.

In isotype-specific competition Western blot, Ab12 inhibited the binding of Ab53 but not of Ab64 to LVS lysate (Figure 2A), suggesting that Ab12 and Ab53 target the same or an overlapping FtGroEL epitope. Because of the lack of inhibition by Ab64, combined with its lower binding to the recombinant proteins seen in Figure 1A left panels, the specificity of Ab64 for FtGroEL was verified by mass spectrometric analysis of tryptic peptides from the gel-isolated antigen following immunoprecipitation by Ab64 from an LVS lysate (Figure 2B).Thus, the competition Western blot demonstrated that Ab64 targets a different FtGroEL epitope than Ab12/Ab53.

An attempt to localize the GroEL epitopes targeted by the Ab53 and Ab64 mAbs using peptide phage-display library screening did not identify any linear GroEL sequences, but computational analysis of the selected peptides suggested possible conformational epitopes that might be validated by mutational analysis. Consequently, several point mutants of FtGroEL in which the amino acids in selected positions were replaced with non-conservative amino acids were generated and expressed as recombinant His-tagged proteins in BL21 *E. coli*. Two of the mutations, a change from lysine to glutamic acid at position 344 (K344E) and a change from tyrosine to glutamic acid at position 476 (Y476E), abolished Western blot binding by Ab64 but not by Ab53.

### Selection of Additional FtGroEL mAbs

**Figure 3:**
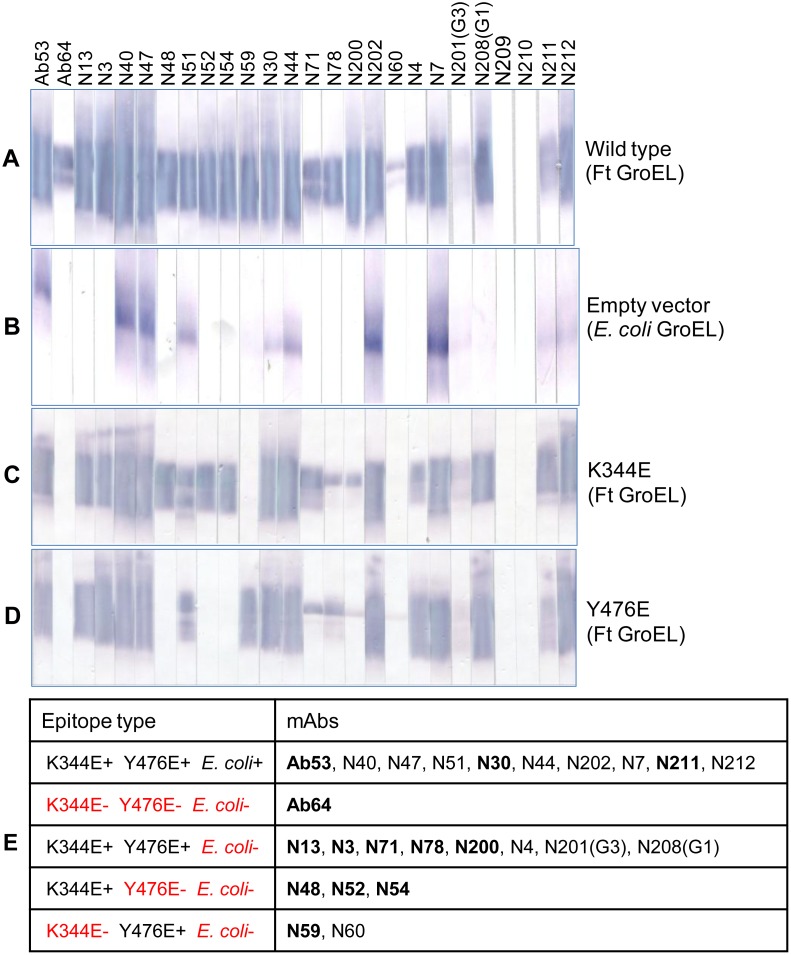
FtGroEL mAbs are classified based on reactivity to FtGroEL, EcGroEL, and FtGroEL mutants. (**A–D**) Western blot. Lysates of *E. coli* transformed with FtGroEL-encoding vector, empty vector, or vectors encoding the K344E and Y476E FtGroEL mutants were electrophoresed in preparative 7.5% polyacrylamide gels under denaturing conditions, allowing the dye in the sample buffer to run out of the gel in an attempt to obtain maximal separation between the Ft and Ec GroEL monomers (hence the stretched-out appearance of the GroEL bands compared with the GroEL bands in the Western blots of Figures 1 and 2). After transfer to nitrocellulose, strips were probed with secondary supernatants of 24 IgG-producing hybridomas or of the Ab53 or Ab64 hybridoma cell lines. (**E**) Classification of FtGroEL mAbs into five epitope types based on Western blot results from A to D. Binding (+) or lack of binding (–) are indicated in black and red font, respectively, and mAbs selected for further analysis are bolded.

The crossreactivity of Ab53 with EcGroEL and lack of reactivity of Ab64 with the K344E and Y476E FtGroEL mutants were exploited for early selection of additional FtGroEL hybridoma mAbs that may target GroEL epitopes other than Ab12/Ab53 and Ab64. Secondary supernatants of 24 IgG-producing hybridomas, whose primary supernatants were suspected of specificity to GroEL based on binding to LVS lysate Western blots, were tested along with Ab53 and Ab64 (a total of 26 mAbs) for Western blot binding to lysates of BL21 *E. coli* transformed with vectors encoding wild-type FtGroEL, or empty vector, or K344E or Y476E FtGroEL mutants. As shown in Figure 3A, 24 of the 26 mAbs tested reacted with the wild type rGroEL, confirming that they are indeed specific for FtGroEL. And 12 of the 24 FtGroEL mAbs (half) crossreacted with EcGroEL (Figure 3B). The large fraction of FtGroEL mAbs that crossreacts with EcGroEL is not surprising in view of the 74% amino acid sequence identity between the two proteins (NCBI Protein Blast http://blast.ncbi.nlm.nih.gov/Blast.cgi). Although the majority of FtGroEL mAbs reacted with both the K344E and Y476E mutants, some reacted only with one of the two or, like Ab64, with neither (Figure 3C and 3D), resulting in a five-group classification (Figure 3E). One to four mAbs from each group (bolded in Figure 3E), all of the IgG2a,κ isotype, were selected for further characterization along with the IgG3,κ mAb Ab12, for a total of 14 mAbs. The hybridomas expressing the selected mAbs were subcloned and one clone of each was expanded and the mAbs were purified and quantified.

### Further Characterization of Selected FtGroEL mAbs

**Figure 4:**
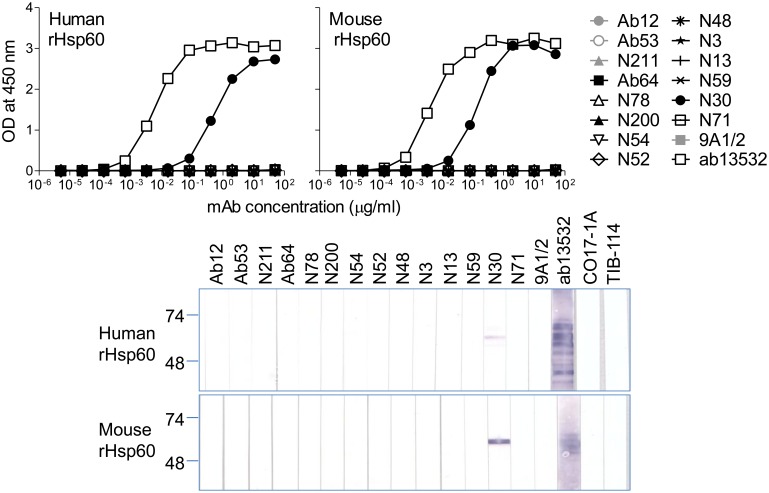
Only one of 14 FtGroEL mAbs crossreacts with human or mouse Hsp60. (**A**) ELISA. Human or mouse recombinant Hsp60 was coated on ELISA plates, and the plates were probed with serial dilutions of the indicated mAbs (made from the same stocks as the ones used in the experiments in Figure 1, except for the ab13532 anti-Hsp60 mAb, which was used only for the experiments in this figure). (**B**) Western blot. Recombinant human and mouse Hsp60 were separately electrophoresed in preparative 4–15% polyacrylamide gels under denaturing conditions and, after transfer to nitrocellulose, strips were probed with 10 µg/ml of the indicated mAbs followed by AP-conjugated-anti-mouse IgG (H+L) (Promega). The positions of prestained molecular weight standards (in kDa) are indicated.

There is considerable sequence homology between bacterial GroELs and mammalian Hsp60 proteins, with 50% identity between FtGroEL and human or mouse Hsp60 (NCBI Protein Blast http://blast.ncbi.nlm.nih.gov/Blast.cgi). Therefore, we tested the 14 FtGroEL mAbs, as well as the 9A1/2 EcGroEL mAb (52% or 53% identity between EcGroEL and human or mouse Hsp60, respectively), for binding to human or mouse rHsp60 in both ELISA and Western blot. As shown in Figure 4, only the N30 mAb bound weakly to human and mouse rHsp60, with 86-fold and 29-fold more mAb required for 50% binding, respectively, than the ab13532 (IgG2a) mAb specific for both human and mouse Hsp60, which was used as positive control. Thus, induction of FtGroEL antibodies that crossreact with mammalian Hsp60 may not be prevalent, and therefore is unlikely to complicate the potential use of FtGroEL as a component of a tularemia vaccine or target of therapeutic antibodies.

Two strategies were used to determine if any of the 14 mAbs bind to the same or overlapping FtGroEL epitopes. First, the nucleotide sequences encoding the variable (V) regions of the 14 antibodies were determined, from which the heavy (H) and light (L) chain V, D and J germline genes of each mAb were deduced by homology searches using the IGBLAST program (http://www.ncbi.nlm.nih.gov/igblast/). As shown in Table 1, Ab12 and Ab53, which were derived from different mice, are encoded by the same IGHV and IGKV genes, and N48, N52 and N54, which were derived from the same mouse, are encoded by the same IGHV, IGHD, IGHJ and IGKV genes. The sharing of germline genes indicates that Ab12 and Ab53 target the same GroEL epitope, and N48, N52 and N54 target the same GroEL epitope.

**Table 1:** Percent nucleotide identity of genes encoding the V regions of FtGroEL mAbs to the most homologous germline genes.

	Germline gene (% nucleotide identity)					
mAb	IGHV	IGHD	IGHJ	IGKV	IGKJ	Mouse No./Immunization
Ab64	1S135*01 (98.4)	1–1*01 (95.2)	4*01 (100.0)	2–137*01 (97.2)	2*01 (100.0)	F3/a
N30	1–9*01 (96.0)	1–1*01 (100.0)	3*01 (100.0)	9–124*01 (94.6)	2*01 (100.0)	176/b
N200	1–18*01 (94.1)	2–14*01 (100.0)	1*01 (98.1)	5–39*01 (98.3)	5*01 (100.0)	190/c
N59	1–69*02 (98.8)	2–14*01 (100.0)	1*01 (100.0)	4–79*01 (98.0)	4*01 (100.0)	171/b
N13	5S9*01 (91.2)	2–5*01 (100.0)	3*01 (97.1)	16–104*01 (98.4)	1*01 (100.0)	179/d
N3	5–17*02 (98.4)	2–3*01 (100.0)	4*01 (97.9)	9–124*01 (92.5)	2*01 (100.0)	187/c
N78	5–17*02 (98.4)	1–2*01 (100.0)	4*01 (98.0)	1–117*01 (98.8)	1*01 (100.0)	184/c
Ab12	6–6*02 (95.0)	**	4*01 (98.0)	1–135*01 (98.1)	1*01 (100.0)	F476/e
Ab53	6–6*02 (98.5)	1–1*01 (100.0)	2*01 (100.0)	1–135*01 (98.1)	2*01 (100.0)	F185/f
N211	6–6*02 (96.2)	2–3*01 (100.0)	2*01 (100.0)	4–57*01 (98.3)	5*01 (100.0)	188/d
N48	2-4-1*01 (97.4)	1–2*01 (100.0)	4*01 (97.9)	6–25*01 (97.1)	4*01 (100.0)	171/b
N52	2-4-1*01 (97.8)	1–2*01 (100.0)	4*01 (97.9)	6–25*01 (97.1)	4*01 (100.0)	171/b
N54	2-4-1*01 (96.1)	1–2*01 (100.0)	4*01 (97.9)	6–25*01 (81.7)	2*03 (97.1)	171/b
N71	14-4*02 (98.4)	4–1*02 (100.0)	2*01 (100.0)	8–27*01 (99.6)	2*01 (100.0)	176/b

**No D gene was identified by the IGBLAST program (http://www.ncbi.nlm.nih.gov/igblast/) using the minimum requirement of five contiguous nucleotides. Shared IGHV, IGHD or IGKV genes are shown in the same color.

a, Antigen extracts of LVS adjuvanted with TiterMax®Gold and CpG ODN 1826 i.p., s.c., i.d. twice, followed by same antigen adjuvanted with CpG ODN 1826 i.p. and i.n., followed by 2×10^3^ and 2×10^4^ LVS i.n.

b, Antigen extracts of the OAg-deficient LVS mutant WbtI adjuvanted with TiterMax®Gold and CpG ODN 1826 i.p., followed by 2×10^5^ or 2×10^7^ LVS i.d., then the same antigen extracts of WbtI i.p.

c, 1.6×10^5^–2.3×10^7^ LVS i.d., followed by antigen extracts of LVS i.p.

d, 1.6×10^5^ or 2.3×10^6^ LVS i.d., followed by antigen extracts of WbtI i.p.

e, 3×10^5^ LVS i.d., 2×10^5^ LVS i.d., 2×10^6^ LVS i.p.

f, 2×10^4^ LVS i.n., followed by FtLPS mAb Ab3 i.p., then 4×10^5^ LVS i.n.

**Figure 5:**
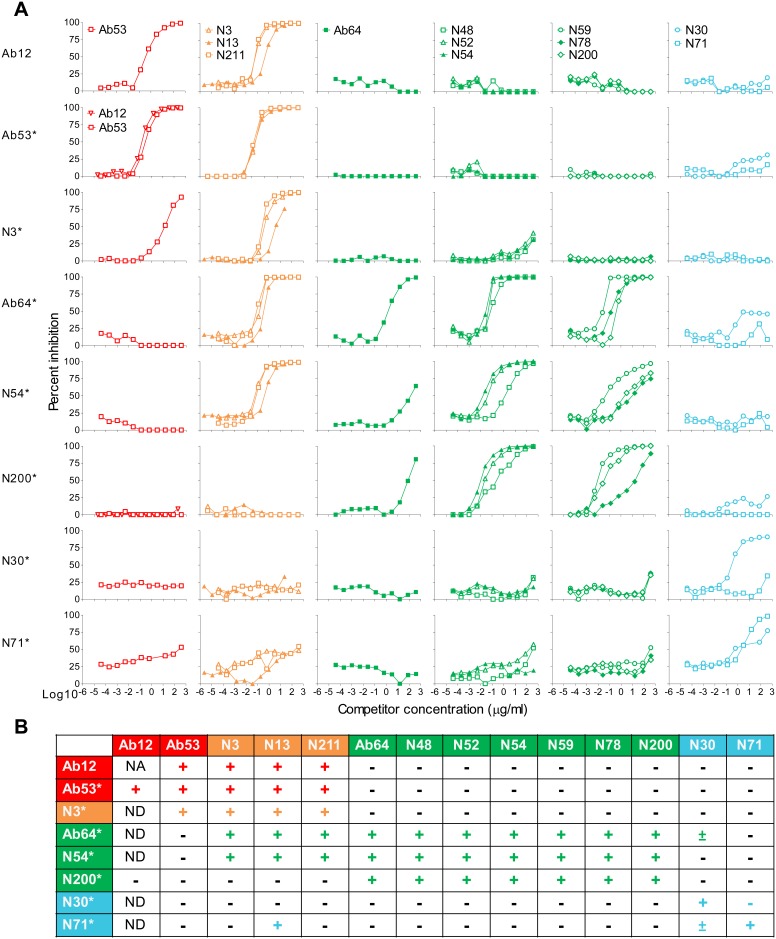
Crosscompetition between 14 GroEL mAbs reveals at least three non-overlapping epitope areas in FtGroEL. (**A**) Competition ELISA. HRP-conjugated streptavidin was used to detect the binding of biotinylated reporter antibodies (indicated by *) to native GroEL (except for Ab12, which was detected by HRP-conjugated anti-mouse IgG3), in the presence of the indicated competitor mAbs. (**B**) Data summary. Competition, defined as a ≥80% increase in percent inhibition is indicated by +; a 40–79% increase in percent inhibition is indicated by ±; and ≤39% increase in percent inhibition is indicated by –. Four groups of mAbs based on similarity in crossblocking profiles are colored red, orange, green or blue. NA, not applicable; ND, not done.

The second strategy compared the ability of the 14 GroEL mAbs to block each other’s binding to FtGroEL in competition ELISA. Because all but one (Ab12) of the 14 GroEL mAbs are of the IgG2a isotype, reporter mAbs were biotinylated and their binding detected with horse radish peroxidase (HRP)-labeled streptavidin. The results of the competition ELISA, shown in Figure 5A and summarized in Figure 5B, revealed seven crossblocking profiles. The 14 mAbs could be divided into four groups based on similarity in crossblocking profile. As expected, Ab12 and Ab53, which share the IGHV and IGKV germline genes, exhibit the same crossblocking profile. N48, N52 and N54, which share the same IGHV, IGHD, IGHJ and IGKV germline genes (Table 1), also exhibit a shared crossblocking profile, which however is distinct from that of Ab53 and Ab12 (Figure 5). Among the mAb pairs in which both members were used as reporters, two pairs did not show reciprocal competition: Ab64 did not inhibit the binding of N3 even though N3 inhibited the binding of Ab64; and N30 showed some inhibition of N71-binding, even though N71 did not show detectable inhibition of N30 binding up to the 400 µg/ml concentration tested (Figure 5A). In both cases, the results could be explained by the weaker binding-potency of the non-competing member of the pair. The four crossblocking profiles define at least three non-overlapping epitope areas, which could be represented by Ab53, Ab64 and N71 or N3, N200 and N30, or Ab53, N200 and N30 (Figure 5B), indicating that at least three different antibodies can simultaneously bind to FtGroEL.

### *In vivo* Efficacy of FtGroEL mAbs

To test the *in vivo* efficacy of FtGroEL mAbs, we used a mouse model of respiratory tularemia in which BALB/c mice are inoculated intranasally (i.n.) with SchuS4 and treated intraperitoneally (i.p.) and/or i.n. with test mAbs, control mAbs, or just phosphate-buffered saline (PBS), the vehicle, 2 hours later. Initial experiments showed no significant prolongation of survival by Ab53 or Ab64, or by the two mAbs combined, compared with PBS alone. This was not surprising because of the very high virulence of Ft SchuS4 in mice, where the LD_100_ is 1 CFU and the mean time to death post i.n. infection is 5–7 days [82]. So far, only mAbs specific for *O*-antigen (of LPS and capsule) have been shown to prolong survival in mice infected i.n. with SchuS4 [31], [33].

**Figure 6:**
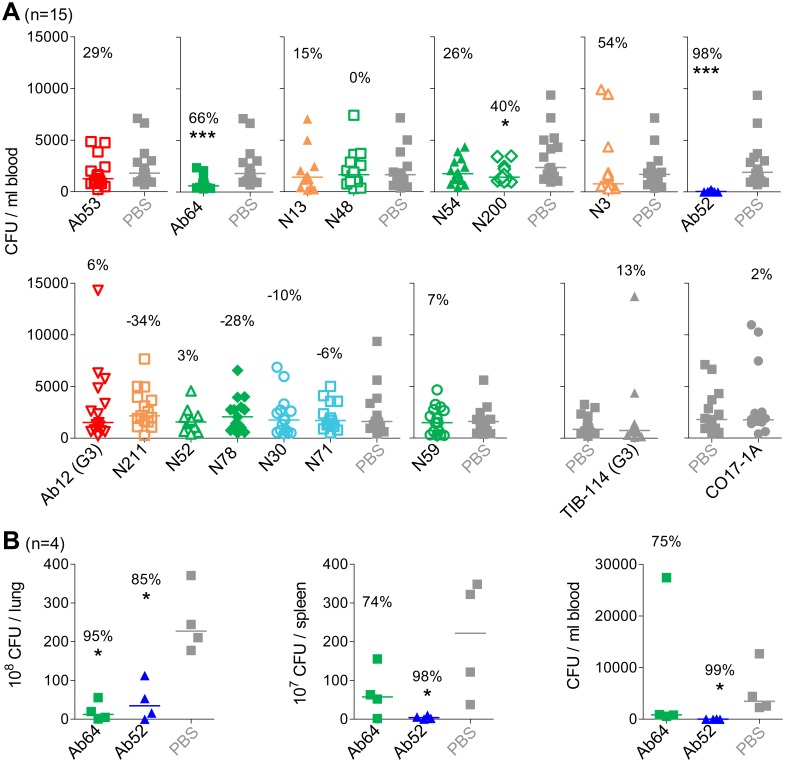
Ab64 and N200 reduce SchuS4 burden in a mouse model of respiratory tularemia. (**A**) BALB/cJ female mice (n = 15) were inoculated i.n. with 91–164 CFU of SchuS4, injected i.p. with 50 µg of the indicated mAbs 2 hours post inoculation, and bled then euthanized 3 days post inoculation for blood CFU determination. Data were pooled from 3 experiments with 5 mice per group for each mAb and compared for statistical significance with PBS only for groups that were tested at the same time (same panel). (**B**) BALB/cJ female mice (n = 4) were inoculated i.n. with 93 (n = 2) or 114 (n = 2) CFU of SchuS4, injected i.p. with 50 µg of the indicated mAbs 2 hours post inoculation, and bled then euthanized 3 days post inoculation for blood, lung and spleen CFU determination. Percent CFU reduction compared with PBS was calculated from the median CFU numbers and the P value (indicated below the percent CFU reduction by *p≤0.05, **p≤0.01, or ***p≤0.001) was determined using the two-tailed Mann-Whitney test. All mAbs are IgG2a except for Ab12 and TIB-114, which are IgG3 (indicated as G3). The Ab52 mAb (anti-Ft O-antigen) was used as standard. The TIB-114 (anti-sheep red blood cells) and CO17-1A (anti-human tumor-associated antigen EpCam) mAbs were used as isotype controls. All other mAbs are anti-Ft GroEL.

To assess the effect of non-*O*-antigen mAbs on the course of SchuS4 infection in the respiratory tularemia mouse model, we used as indicator of efficacy reduction in blood bacterial burden 3 days post infection, which we had previously shown, using Ft *O*-antigen mAbs, to correlate with delayed time-to-death [31]. Of the 14 GroEL mAbs, only Ab64 and N200, which have the same crossblocking profile (Figure 5B) and whose binding to GroEL is abolished or greatly diminished by the K344E and Y476E mutations (Figure 3), significantly reduced blood bacterial burden compared with PBS, by 66% and 40%, respectively (Figure 6A). No synergism in reduction of blood bacterial burden was observed with an equimolar mixture of Ab64 and Ab53 (data not shown). But Ab64 alone also caused significant reduction of bacterial burden (95%) in the lungs of SchuS4-infected mice (Figure 6B). These results indicate that the FtGroEL epitope(s) targeted by the crossblocking mAbs Ab64 and N200 are protective and suggest that the protective effect of GroEL antibodies against type A Ft may not be due simply to opsonization or complement activation, in which case the efficacy of mAbs of the same IgG subclass (13 of the 14 are IgG2a) would be expected to correlate directly with their bivalent avidity for antigen. Instead, as gleaned from the ELISAs and Western blots in Figures 1–3 and other experiments in our laboratory, Ab64 is one of the weakest GroEL-binding mAbs and many of the other GroEL-binding mAbs are at least as potent as N200. This suggests that Ab64 and N200 may exert either a direct protective effect by neutralizing a pathogenic function of FtGroEL or an indirect protective effect by inducing or stabilizing a conformational change in FtGroEL, leading to immune activation. Support for the latter possibility comes from the report that FtGroEL synergizes with Ft LPS to induce secretion of the CXCL8 chemokine by human monocyte-derived macrophages [71].

### FtGroEL Homology Model and Mapping of Epitopes Targeted by FtGroEL mAbs

As already mentioned, our initial attempt to map the epitopes targeted by the Ab53 and Ab64 mAbs by peptide phage display library screening (see File S1) did not identify any continuous FtGroEL sequences. To determine whether the peptides selected by the two mAbs (Table S1 in File S1) could reveal conformational epitopes, we constructed a homology model of FtGroEL based on the published X-ray crystal structure of EcGroEL [42]. In this structure, the EcGroEL tetradecamer is an asymmetric dimer, where one of the seven-member EcGroEL rings is saturated with bound ADP and interacts with seven EcGroES chains, which form a cap on top of the ring. The conformations of the EcGroEL monomers in the GroES-bound ring and the non-GroES-bound ring differ [42].

**Figure 7:**
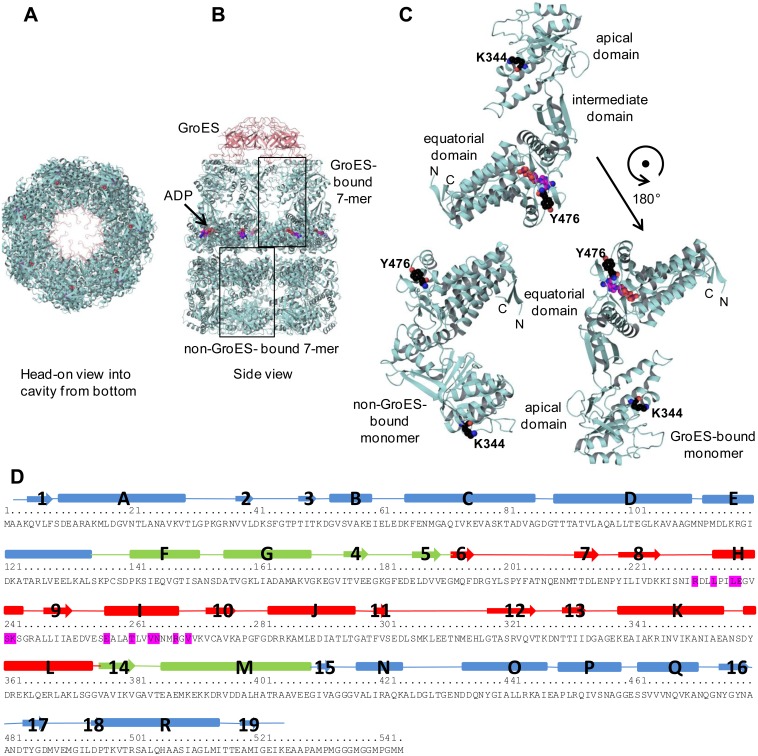
Homology model of FtGroEL. (**A–C**) Ribbon diagrams of GroEL tetradecamer complexed with GroES and of the non-GroES-bound and GroES-bound monomers. The positions of ADP (space-filling model in purple), of FtGroEL residues K344 and Y476 (space-filling models), and of the amino (N) and carboxyl (C) termini of the monomers are indicated. The GroES-bound monomer in C top is also shown after a 180° rotation about the z axis in C bottom right, to facilitate comparison with the non-GroES-bound monomer in C bottom left. (**D**) Linear amino acid sequence (in one-letter code) and secondary structure representation of FtGroEL. α-helices, β-strands, and loops are represented as boxes, thick arrows, and lines, respectively, blue for the equatorial domain, green for the intermediate region, and red for the apical domain. Helix letters and strand numbers are indicated. The FtGroEL amino acid residues involved in the interaction with GroES are highlighted in magenta.

The FtGroEL model was built based on the bullet-shaped structure of the EcGroEL/EcGroES/ADP complex (Protein Data Bank http://www.rcsb.org/pdb/home/home.do code 1AON). The FtGroEL SchuS4 sequence (NC_006570 REGION: 1764262.1765896 [83]), which has 74% sequence identity to EcGroEL with no gaps or deletions (http://blast.ncbi.nlm.nih.gov/Blast.cgi), was threaded onto the GroES-bound chain and non-GroES-bound chain of the 1AON structure, representing each conformation of EcGroEL observed in the crystal structure. Each seven-member ring was then generated by overlay of the GroES-bound and non-GroES-bound FtGroEL models onto the other chains from the corresponding ring in the EcGroEL structure. FtGroES and EcGroES have 57% sequence identity and there is a single residue gap between the sequences. To create the seven-member GroES ring, the FtGroES model was superposed onto the other chains of EcGroES in the 1AON structure. The initial FtGroEL/FtGroES model was manually inspected and all Ft-unique residues were found to fit well into the structure with some minor rotamer (side-chain conformation) changes. Figure 7A–C shows head-on and side views of the FtGroEL/FtGroES homology model and side views of the GroES-bound and non-GroES-bound FtGroEL monomers as ribbon diagrams. The GroES-bound monomer is also shown after a 180° clockwise rotation about the z axis (Figure 7C bottom right), to facilitate comparison with the non-GroES-bound monomer (in Figure 7C bottom left). Each monomer consists of an equatorial domain (amino acid residues 1–134 and 410–525, comprised of α-helices A-E and N-R) and an apical domain (residues 191–375, comprised of a β-sheet flanked by α-helices H-L) connected by an intermediate region that consists of three β-strands and three α-helices (shown in ribbon diagram in Figure 7C and in linear diagram corresponding to the amino acid sequence in Figure 7D). In the GroES-bound GroEL, the interaction with GroES (Figure 7B) involves 12 residues in helices H and I (Figure 7D, residues highlighted in magenta). The different conformations of the apical domains, and to a lesser extent of the equatorial domains, in the non-GroES-bound and GroES-bound monomers is reflected in the shifted positions of FtGroEL apical residue K344 and equatorial residue Y476 (Figure 7C).

The FtGroEL homology model was evaluated using computational programs in an attempt to discover the antigen surface mimicked by the peptides selected in the peptide-phage-display-library screening, thereby yielding putative epitopes for each Ab53 and Ab64 (see File S2). To validate these putative epitopes, several point mutants of FtGroEL, in which residues in the putative Ab53 or Ab64 epitopes were substituted by those expressed in other bacteria or by charged residues (Figure S1 in File S2), were generated and recombinantly expressed in *E. coli* (see Figure S2A in File S3). As already discussed, two of the mutations, K344E made for Ab53 and Y476E made for Ab64, abolished binding of Ab64 to the FtGroEL monomer on Western blots but did not affect the binding of Ab53 (Figure S2B in File S3), thereby failing to validate the computationally-predicted epitopes. Nonetheless, as described above, the K344E and Y476E mutations were successfully exploited for early selection of diverse FtGroEL mAbs, revealing that each of these mutations abrogates or reduces binding of Ab64 and N200, respectively, to FtGroEL (Figure 3C and 3D).

To map the Ab53 and Ab64 epitopes, as well as those targeted by three other FtGroEL mAbs of interest (N200, N3 and N30) based on *in vivo* efficacy and competition ELISA data, we used hydrogen/deuterium exchange - mass spectrometry (DXMS), which exploits the continuous reversible exchange of peptide-amide hydrogens in proteins with water hydrogens [84], [85]. The exchange rate of each hydrogen correlates directly with the extent to which it is exposed (accessible) to solvent [84], [85], and antibody binding to a protein antigen slows the exchange rates in the antigen segments that contact the V regions of the antibody (the epitope-containing segments). The exchange rates for free and antibody-bound antigen are determined by incubating each in buffer with deuterated water (D_2_O) for graded time periods followed by proteolysis into overlapping peptides, which are separated chromatographically. The deuterium content of each peptide is then analyzed by mass spectrometry to obtain a ‘heat-map’ of the exchange rate for the entire protein antigen. The sequences(s) of the antigen in which deuterium exchange was inhibited by antibody-binding are revealed by subtracting the DXMS heat-map of the antibody-bound antigen from the DXMS heat-map of the free antigen to obtain a ‘difference heat-map’, in which the darkest blue region(s) identify the epitope. Although the spatial resolution of DXMS is not at the single residue level, the antigen segments comprising the epitope are localized to within a few amino acids [85], and are referred to as ‘epitopes’ in the context of DXMS studies.

**Figure 8:**
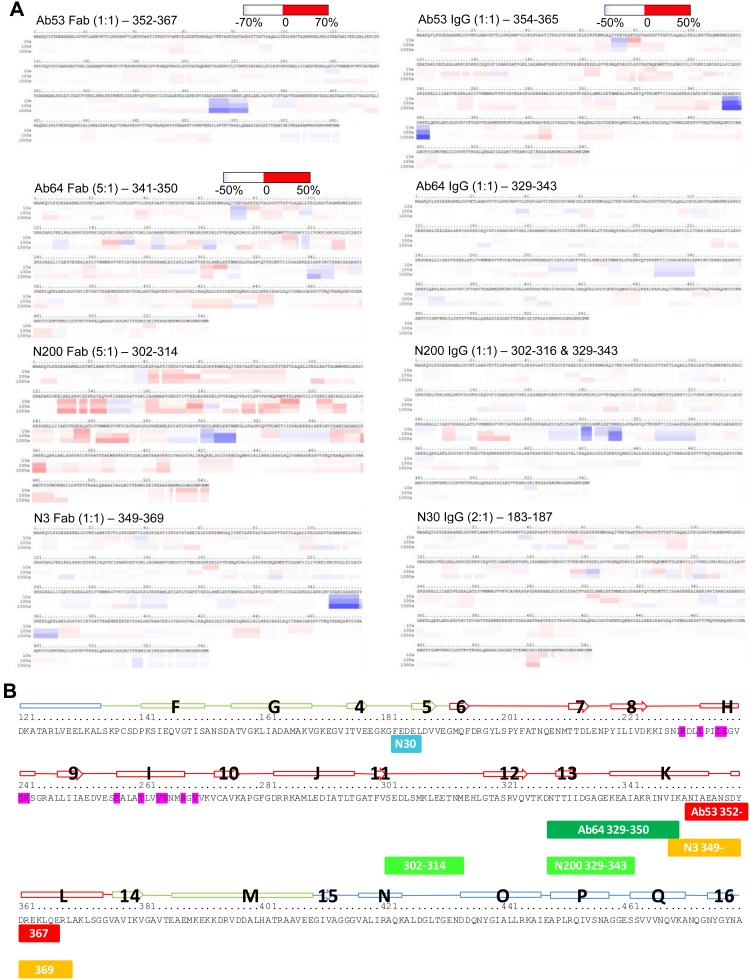
DXMS-mapping of mAb epitopes in FtGroEL. (**A**) Difference heat-maps for FtGroEL complexed with Fab (left) or IgG (right) of the indicated mAbs in the indicated molar ratio of mAb to FtGroEL monomer. The times of FtGroEL-Ab interaction (10 seconds, 100 seconds, and 1000 seconds) are specified. As indicated in the color bar, blue shades suggest more buried regions (which exchange slower upon antibody-binding), and red shades suggest more exposed regions (which exchange faster upon antibody-binding). The darkest blue region(s) in each difference heat-map were taken as the DXMS-epitopes and their spans are indicated. Note that the heat map for FtGroEL in complex with Ab53 Fab (top left, the first one to be performed) is in a slightly different format than the other heat-maps. (**B**) Linear representation of the DXMS-epitopes on the sequence/secondary structure template of FtGroEL (see legend to Figure 7D for description, except partial view). When both Fab and IgG data were available, the results were combined. Epitopes for the indicated mAbs are represented as labeled colored boxes below the FtGroEL sequence.

Fab, intact IgG, or both were used in attempts to DXMS-map the epitopes of the five mAbs. The DXMS heat-maps of free FtGroEL and Fab-bound or IgG-bound FtGroEL followed by the difference heat-map are presented in Figure S4 in File S4. Reduced-size versions of the difference heat-maps are also shown in Figure 8A, and the epitopes are diagrammed in Figure 8B. The epitope regions were more clearly identified for Ab53, N200 and N3 than for the weaker-binding Ab64 and N30 (Figure 8A). Of the three mAbs for which both Fab and IgG were used (Ab53, Ab64 and N200), the Ab53 results indicate the same continuous epitope region with both Fab and IgG, whereas the Ab64 and N200 results indicate different but overlapping epitope regions with Fab and IgG (Figure 8A). In these cases, the Fab and IgG results for each epitope were combined (Figure 8B). The Ab53 and N3 DXMS epitopes overlap, with the smaller Ab53 epitope contained within the N3 epitope. Thus, the larger N3 epitope but not the Ab53 epitope has a 2-amino acid overlap with the Ab64 epitope (Figure 8B), consistent with the ability of N3 but not Ab53 to inhibit the binding of Ab64 to FtGroEL (Figure 5). The Ab64 and N200 epitopes overlap partially, with the N200 epitope comprised of two segments separated by 14 amino acids and the Ab64 epitope extending past the N200 epitope to overlap with the N3 epitope by two amino acids (Figure 8B). These relative locations of the Ab64, N200 and N3 epitopes are consistent with the ability of N3 to inhibit the FtGroEL-binding of Ab64, as well as the inability of N3 and N200 to crossblock each other’s binding to FtGroEL (Figure 5). For N30, the DXMS results indicate an epitope region spanning amino acids 183 to 187 (Figure 8). None of the DXMS epitopes overlaps with the binding site of GroES on FtGroEL (Figure 8B), indicating that none of the mAbs are likely to interfere with the GroES-GroEL interaction.

**Figure 9:**
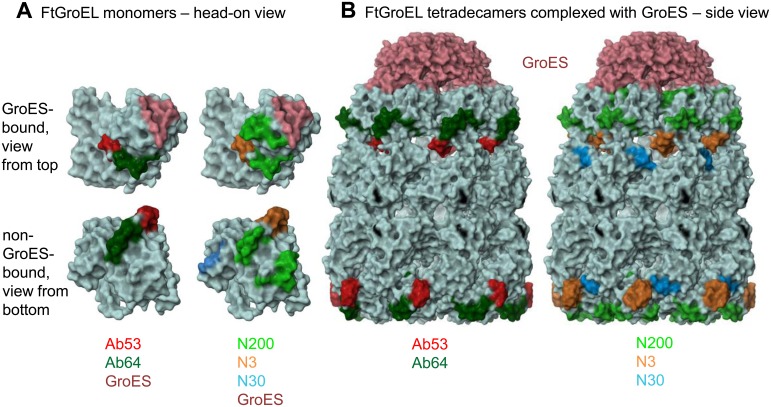
DXMS-epitopes of FtGroEL mAbs on the molecular surface of Ft GroEL-GroES homology models. (**A**) FtGroEL monomers. (**B**) FtGroEL tetradecamers complexed with GroES. The epitopes of the five mAbs and the binding-site of GroES on GroEL are color-coded as indicated, and are divided into two images to separate the overlapping epitopes. FtGroEL residue Y476 is indicated in black. Note that the N30 epitope is exposed on both the GroES-bound and non-GroES-bound monomers but is blocked from view by the apical domain in the head-on view of the former. The proximity of the N3 and N30 epitopes in the tetradecamer, especially in the non-GroES-bound heptamer, reflects the juxtaposition of epitopes from neighboring monomers.

The DXMS epitopes of the five FtGroEL mAbs are indicated on the molecular surface of the homology models of the non-GroES-bound and GroES-bound FtGroEL monomers, in two groups of non-overlapping epitopes (Ab53 and Ab64; and N200, N3 and N30; the binding site of GroES on FtGroEL is also shown on the GroES-bound monomers; Figure 9A). The models indicate that the DXMS epitopes contain solvent-exposed residues that should be accessible to antibodies. However, whereas the Ab64, N200 and N30 epitopes are fully surface-exposed both on the non-GroES-bound and GroES-bound heptamers, the part of the Ab53 and N3 epitopes formed by helix L and the preceding loop (see Figure 8B) moves from the outside of the heptamer ring to the inside of the ring, forming part of the wall of the folding chamber (Figure 9B). Thus, the Ab53 and N3 epitopes are only partially surface-exposed on the GroES-bound heptamer ring. The fraction of FtGroEL tetradecamers containing a GroES-bound heptamer in the purified FtGroEL used in the DXMS experiments is not known. As Ab53 and/or N3 may not be able to bind to the exposed parts of their epitopes on the GroES-bound heptamer, it is possible that some of the mAbs bind to both heptamer rings whereas others bind only to one. Nonetheless, the surface-exposure of all five DXMS epitopes on one or both heptamer rings of the FtGroEL homology model provides support for its validity. Furthermore, the ability of Ab64 and N200, but not of Ab53 and N3, to interact with the GroES-bound heptamer may account for the ability of the former but not the latter pair to reduce blood bacterial burden in the mouse model of respiratory SchuS4 infection (Figure 6). In the case of N30, although the DXMS-mapped residues 183–187 are partially in strand 5, which is at the end of the intermediate domain, it is possible that the full epitope spans domains and includes residues in the apical domain that are not properly oriented for binding to the antibody in the GroES-bound GroEL conformation. The GroES binding-site does not overlap with any of the five DXMS epitopes, coming close to only part of the N200 epitope (Figure 9), suggesting that both GroES and each of the five antibodies can be bound to FtGroEL simultaneously.

The DXMS results also explain the sensitivity of both Ab64 and N200 to the K344E mutation because both epitopes contain or extend up to the K344 residue (Figure 8). The reason for the sensitivity of the two mAbs to the Y476E mutation is not immediately clear, since Y476 is in the equatorial domain of FtGroEL (Figure 7C and 7D, and highlighted in black in Figure 9B), far from the two epitopes in the apical domain. It is possible though that the Y476E mutation exerts an allosteric effect on the structures of the Ab64 and N200 epitopes, especially given the position of this residue close to the junction of the two FtGroEL heptamer rings. This possibility is supported by the rearrangement induced in the apical domain by the binding of ATP to the equatorial domain [42] and the ability of the Ab64 and N200 mAbs to bind to the GroES-bound heptamer. The crossreactivity of Ab53 with EcGroEL (Figure 3B) is consistent with the 88% (14/16) identity between the Ft and Ec sequences in the DXMS epitope of Ab53 compared with 74% between the Ft and Ec entire GroEL proteins, respectively (http://blast.ncbi.nlm.nih.gov/Blast.cgi). It is noteworthy that N3 does not crossreact with EcGroEL (Figure 3B) despite the Ab53 DXMS epitope being contained within the N3 DXMS epitope (Figure 8). A likely explanation is that the FtGroEL residues that actually contact the V regions of the antibody differ between Ab53 and N3, and/or that minor Ft/Ec sequence differences within or outside the N3 epitope cause substantial differences in local structure between FtGroEL and EcGroEL.

### X-Ray Crystal Structures of Ab53 and Ab64 Fabs

**Figure 10:**
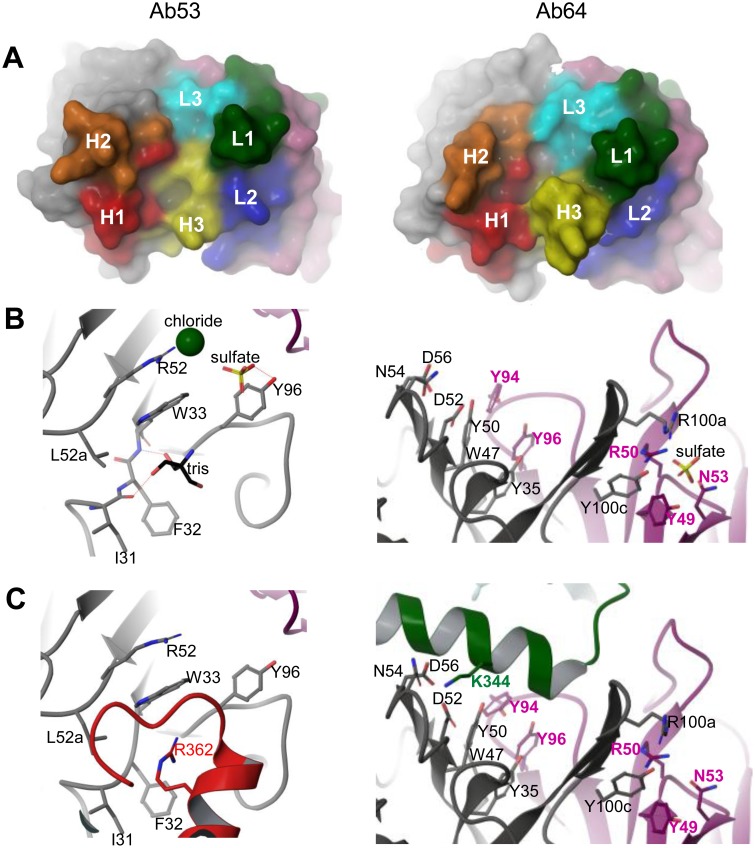
X-ray crystallographic structures of the antigen-binding sites of Ab53 and Ab64. (**A**) Head-on views of the molecular surfaces, colored gray for the H chain and purple for the L chain, with the CDR loops colored and indicated as H1, H2, H3 for the H chain and L1, L2, L3 for the L chain. (**B**) Ribbon diagrams of the binding-sites, colored gray for VH and purple for VL, and clipped/depth-cued for clarity. Selected side chains are shown in stick and labeled in black for VH residues and purple for VL residues. Solvent molecules present in the structures (sulfate, chloride, tris) are also shown. Hydrogen bonds indicated in the text are shown as dotted lines. (**C**) Ribbon diagrams of the binding-sites with the docked FtGroEL model, shown in red for Ab53 with the side-chain of R362 and in green for Ab64 with the side-chain of K344. The positions of some of the residues in the docked antibody structures are somewhat shifted compared with the crystal structures in B due to the energy minimization steps used during the docking protocol. Figure made with Maestro (version 9.3.5, Schrödinger, Inc., New York, NY).

Further validation of the FtGroEL homology model was obtained by determining the X-ray crystal structures of Ab53 and Ab64 Fabs at 2.5 and 2.6 Å resolution, respectively. Both structures have good geometry and agreement to the observed X-ray diffraction data (Table 2). The final Ab53 structure contains H chain residues 1–212 (with the exception of residues 128–133, which could not be modeled due to poor electron density), L chain residues 1–211, 186 water molecules, 2 sulfate ions, 6 chloride ions and one tris molecule. Analysis of the antigen-binding site of Ab53 shows an open-ended groove, 26–30 Å long and 10–20 Å wide, with walls formed by the HCDR1, HCDR2 and LCDR1 loops (H1, H2 and L1), and several pockets in the floor of the groove (Figure 10A left). One tris, one chloride, and one sulfate molecule are bound in the putative antigen-binding site, where they may be making interactions similar to those of the FtGroEL amino acids in the epitope (Figure 10B left). The sulfate ion makes a hydrogen bond to Y96 in the H chain (H-Y96). The chloride ion makes a salt bridge with the side chain of H-R52 (Figure 10B left). As the Ab53 DXMS-epitope contains five Asp or Glu residues (E355, D359, D361, E363 and E367, Figure 8B), the sulfate and chloride ions may be mimicking carboxyl groups of these amino acids. The tris molecule is located in a pocket formed by H-I31, H-F32, H-W33, H-L52a, H-G95, and H-Y96, and makes hydrogen bonds with the backbone atoms of H-I31 and H-W33. Additionally, H-W33 may participate in cation-π interactions with the tris molecule. There are two positively-charged amino acids in the Ab53 DXMS-epitope, R362 and K364 (Figure 8B), and the tris molecule may mimic side-chain interactions of these FtGroEL residues.

**Table 2:** Data collection and refinement statistics for Ab53 and Ab64 Fabs.

Parameter	Value	
	Ab53	Ab64
**Data collection**		
Space group	P6_1_22	R3
Unit cell dimensions	a = b = 151.827 Å, c = 102.770 Å, α = β = 90°, γ = 120°	a = b = 154.571 Å, c = 58.264 Å, α = β = 90°, γ = 120°
Resolution (Å)	15–2.5 (2.59–2.50)	15–2.6 (2.69–2.6)
Number of reflections	23634 (2310)	15858 (1569)
Completeness (%)	96.3 (96.0)	98.8 (97.5)
Average I/σI	22.1 (5.0)	29.0 (5.3)
R_merge_	0.071 (0.321)	0.049 (0.339)
Redundancy	6.8 (6.0)	5.8 (5.7)
**Refinement**		
R	0.2097	0.1852
R_free_	0.2517	0.2397
Non-solvent atoms	3307	3326
Solvent atoms	215	39
R.m.s.d. ideal bonds (Å)	0.002	0.004
R.m.s.d. ideal angles (°)	0.619	0.739
**Ramachandran Plot**		
Favored (%)	97.41	95.05
Allowed (%)	2.59	4.48
Outliers (%)	0.00	0.47

The final Ab64 structure contains H chain residues 1–213 (except for residues 128–133), L chain residues 1–214 (except for residues 152–154), 34 waters, and one sulfate. The Ab64 antigen-binding site is a shallow groove, 24–32 Å long and 9–19 Å wide, walled by H2, H3 and L1 (Figure 10A right), and the floor formed by L3, H1 and part of H2. The sulfate ion is located near H-R100a and H-Y100c from the HCDR3 loop, and L-Y49, L-R50 and L-N53 from the LCDR2 loop, away from the putative antigen-binding site (Figure 10B right), so is unlikely to mimic an interaction of FtGroEL with the antibody. The Ab64 antigen-binding site is made up of mostly polar, uncharged residues, including seven tyrosine residues, four of which (L-Y94, L-Y96, H-Y35 and H-Y50) are clustered near H-W47 (Figure 10B right).

### Experimentally-Validated Computational Docking of the X-ray Crystal Structures of Ab53 and Ab64 Fabs onto the Homology Model of FtGroEL

To obtain models of the Ab53 and Ab64 Fabs in complex with FtGroEL, the two crystal structures were computationally docked onto the homology model of the non-GroES-bound FtGroEL monomer. Docking calculations can be greatly increased in accuracy by incorporating binding-site information obtained from other techniques in the selection of the correctly docked pose in the computational program (ClusPro 2.0 in the current study). This practice is referred to as ‘experimentally-validated computational docking’ (reviewed in [86]). Thus, DXMS epitope mapping, mutational data, and competition ELISA data were used to select the final docked models. To account for the DXMS results (Figure 8), attractive terms were added to residues 352–367 in Ab53 and 329–350 in Ab64. After docking, contact residues of the antigen (residues within 4 Å of the antibody, a distance used by the Molecular Modeling Database to define contacts [87], [88]) were determined for the top 25 output poses from ClusPro. All poses included the residues in the DXMS epitopes. The poses were then analyzed to find the ones that had the most contact residues within the DXMS epitope and the fewest contact residues that showed no change or an increase in hydrogen-deuterium exchange rate.

**Figure 11:**
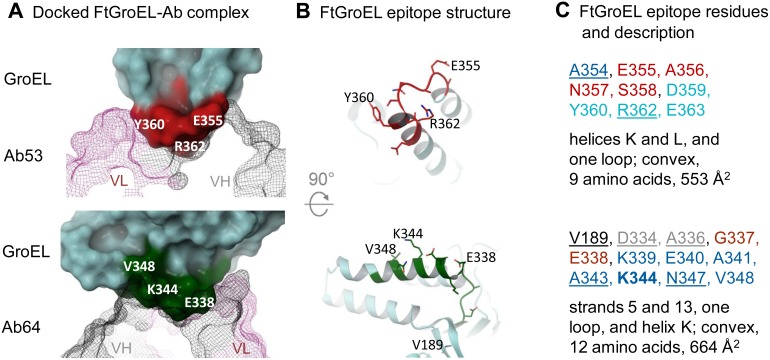
Docked complexes of FtGroEL with Ab53 or Ab64 Fab reveal the FtGroEL contact residues. (**A**) Docked complexes. FtGroEL is shown as a solid molecular surface in cyan with contact residues colored red for Ab53 or green for Ab64. Ab V regions are shown as a wire-mesh molecular surfaces colored grey (VH) and purple (VL). Selected contact residues are indicated in each panel for reference. (**B**) FtGroEL epitope structures with stick side-chains for the reference indicated contact residues as well as V189, which is in the back in the view shown in A and therefore cannot be seen. (**C**) FtGroEL epitopes and description. The list of contact residues was annotated as in [86]. The first discontinuous residue in each epitope segment is underlined. Residues critical to Ag-Ab binding, determined by analysis of point mutants, are bolded. Secondary structure or sugar chain is indicated by color code, with two alternating hues of the same color denoting independent structural elements: black/gray, β-strand; blue, α-helix; red/magenta, loop; green, sugar chain. The surface areas (SA) of the FtGroEL epitopes were calculated from the models using the PISA server [113].

The results of the dockings are shown in Figure 10C as ribbon diagrams and in Figure 11A as solid molecular surfaces of the FtGroEL epitopes complexed with wire-mesh molecular surfaces of the mAb V regions. In addition, ribbon diagrams of the FtGroEL contact residues (the epitopes) are shown in Figure 11B, and annotated listings plus descriptions of the epitopes [86] are shown in Figure 11C. For Ab53, 9 out of 16 DXMS-mapped residues are in contact with the antibody, and these are the sole contact residues observed in the model (Figure 11 top). Seven of the 9 contact residues are conserved with EcGroEL, consistent with the crossreactivity of Ab53 with EcGroEL (Figures 1 and 3B). Additionally, the side chain of R362 of FtGroEL in the docked complex occupies the same space as the positively charged tris molecule observed in the crystal structure, where it makes an interaction with the side-chain of Ab53 H-W33 (Figure 10C left). Only three of the 9 contact residues are conserved with human and mouse Hsp60, consistent with the lack of crossreactivity of Ab53 with the two mammalian proteins (Figure 4).

For Ab64 (Figure 11 bottom), 11 out of 22 DXMS-mapped residues are in contact with the antibody, with only one residue outside the DXMS epitope making a hydrogen bond (V189, whose deuterated status is not determined in the DXMS assay, see Figure S4 in File S4). The proximity of V189 to the 183–187 epitope region of N30 suggested by the DXMS results (Figure 8) explains the ability of N30 to partially inhibit the binding of Ab64 to FtGroEL (Figure 5). Of the 12 FtGroEL residues making contacts to the antibody in the docked model, only 5 are conserved in EcGroEL. The non-conserved residues include changes predicted to preclude binding of Ab64 to EcGroEL, such as FtGroEL K339, which is Glu in EcGroEL. This is consistent with the observation that Ab64 does not bind to EcGroEL (Figures 1 and 3B). Only three of the 12 contact residues are conserved with human and mouse Hsp60, also consistent with the lack of crossreactivity of Ab64 with the two mammalian proteins (Figure 4). The FtGroEL contacts include 6 polar residues, a finding that was expected given the polar nature of the Ab64 antigen binding-site. In the docked model, K344 contacts the antibody via the lysine side-chain, which sits in a pocket formed by antibody residues H-D52, H-N54, and H-D56 (Figure 10C right). The mutational analysis showed that substituting a glutamate at the K344 position of FtGroEL abolishes binding of Ab64 to FtGroEL (Figure S2B in File S3). This observation is consistent with the Ab64-FtGroEL docked model since the glutamate would not be expected to make favorable interactions in this negatively-charged pocket. Inspection of the contact residues shows none in common with Ab53, but two (N347 and V348) in common with the N3 DXMS-epitope (Figure 8), suggesting that Ab64 would compete with N3 but not with Ab53 for binding to FtGroEL, consistent with the competition ELISA data (Figure 5).

Based on the models of the docked FtGroEL-mAb complexes, both the Ab53 and Ab64 epitopes are convex and helical, spanning 553 and 664 Å^2^, respectively (Figure 11C). However, whereas all 12 Ab64 contact residues are outer-surface-exposed in both the non-GroES-bound heptamer and the GroES-bound heptamer, only 4 of 9 Ab53 contact residues are outer-surface-exposed in the GroES-bound heptamer (Figure 9B), making it unlikely that Ab53 can bind to the GroES-bound heptamer. The likely interaction of Ab64, but not of Ab53, with the GroES-bound ring, especially in bivalent IgG form, may prevent, induce, or stabilize a conformational change in FtGroEL, which may account for the protective effect of Ab64 but not Ab53 in the mouse model of respiratory SchuS4 infection (Figure 6). This hypothesis is supported by the difference in the Fab and IgG DXMS profiles of FtGroEL with Ab64 and N200 but not with Ab53 (Figure 8A), since the bivalent IgG interaction may be required for such influence on the FtGroEL structure. The *in vivo* efficacy of Ab64 also would be explained if Ab64 bound with higher affinity to GroES-bound GroEL than to non-GroES-bound GroEL. The former is expected to be much more prevalent *in vivo* than in the purified *in vitro* form, which is not expected to retain or to capture the ATP necessary for the conformational rearrangement that leads to interaction with GroES.

### Concluding Remarks

Although FtGroEL was previously tested as a vaccine candidate in a mouse model of tularemia, protection against Ft LVS and the non-attenuated type B strain HN63 was attributed, at least in part, to contamination of the GroEL preparation with Ft LPS [77]. In the current study, the use of mAbs which, by definition, are not contaminated with antibodies of other specificities, allowed the unambiguous demonstration of protective FtGroEL B-cell epitopes in the mouse model, indicating that FtGroEL is a protective B-cell antigen. FtGroEL may also be a protective T-cell antigen, in view of the recent report of isolation of a mouse T-cell clone specific for an FtGroEL epitope [76]. Whether FtGroEL is both a protective B- and T-cell antigen, our results suggest that it should be evaluated as a component of a subunit Ft vaccine and as a target of antibody immunotherapy for tularemia.

## Materials and Methods

### Bacterial Strains

*F. tularensis* holarctica strain LVS was obtained from Dr. Jeannine Petersen (Centers for Disease Control and Prevention, Fort Collins, CO) and was manipulated under biosafety level 2 (BSL2) containment conditions. *F. tularensis* strain SchuS4 was obtained from BEI Resources, Manassas, VA in accordance with all federal and institutional select agent regulations. All manipulations of SchuS4 were conducted under BSL3 containment conditions. *Escherichia coli* strain TG1 was purchased from Stratagene (La Jolla, CA). Bacterial cultures were grown as previously described on chocolate agar plates at 37°C (for LVS and TG1) or 35°C (for SchuS4) for 2.5 days. Bacteria were scraped and re-suspended in PBS. Bacterial NP40 lysates were generated as described [32].

### Mouse Monoclonal Antibodies

The TIB-114 hybridoma, producing an IgG3 mAb against sheep red blood cells, was obtained from the American Type Culture Collection (ATCC, Manassas, VA). Hybridoma cell line CO17-1A, producing an IgG2a mAb specific for the human tumor-associated antigen EpCam [89], [90], was obtained from Dr. Dorothee Herlyn of the Wistar Institute (Philadelphia, PA). Protein G-purified IgG1 mAb 9A1/2, specific for EcGroEL, and IgG2a ab13532, specific for both human and mouse Hsp60, were purchased from Abcam Inc. (Cambridge, MA). The Ab58 IgG1 hybridoma mAb, specific for histone-like protein HU form B, was generated in our laboratory by fusion of Sp2/0 cells with splenocytes from a BALB/cJ mouse immunized with an antigen extract of LVS, and its target antigen was identified by mass spectrometry as described below. The Ab52 and FB11 IgG2a hybridoma mAbs, specific for Ft LPS, were generated in our laboratory [91] or purchased from GeneTex Inc. (Irvine, CA), respectively.

We have previously described the Ab12 hybridoma, producing an FtGroEL IgG3 mAb, which had been generated from LVS-infected mice [32]. Twenty four additional FtGroEL hybridomas were generated in this study by fusion of spleen cells from immunized BALB/cJ female mice (Jackson Laboratory, Bar Harbor, Maine) with the Sp2/0-Ag14 mouse myeloma cell line [92] as previously described [32]. All animal studies have been reviewed and approved by the Boston University Medical Center Animal Care and Use Committee. Immunizations were done by intradermal (i.d.) or intranasal (i.n.) infection with 2×10^3^–2×10^7^ CFU of LVS, preceded and/or followed by intraperitoneal (i.p.), subcutaneous (s.c.), i.d., and/or i.n. immunization with antigen extracts of LVS or the OAg-deficient LVS mutant WbtI, adjuvanted with TiterMax Gold (CytRx Corporation, Norcross, GA) or CpG ODN 1826 (TCC ATG ACG TTC CTG ACG TT) (an oligodeoxynucleotide containing unmethylated CpG dinucleotides with fully phosphorothioated backbone) (Hycult Biotech Inc. Plymouth Meeting, PA). For one of the hybridomas (Ab53), the mouse was treated i.p. with the Ft LPS mAb Ab3 [32], preceded and followed by i.n. infection with LVS. Fusions were performed 3.75 days after the last immunization, and the isotypes of new hybridomas were determined by ELISA using the Mouse MonoAb ID Kit (HRP) from Zymed Laboratories (South San Francisco, CA) or by IsoStrip (Mouse Monoclonal Antibody Isotyping Kit, Roche Diagnostics, Indianapolis, IN).

For purification of mAbs, hybridoma cells were cultured in IMDM (GIBCO, Grand Island, NY) supplemented with 10% FBS and grown to mass culture in IMDM supplemented with 2% FBS in 10-cm OPTILUX petri dishes (Becton Dickinson Labware, Franklin Lakes, NJ) or in a CELLine *classic* 1000 two-compartment bioreactor (Wilson Wolf Manufacturing, New Brighton, MN) at 37°C in a humidified environment of 5% CO_2_/95% air. mAbs were separately purified from culture supernatants on PIERCE Protein G (IgG1, IgG2a, IgG2b) Plus or Protein A (IgG3) Plus Agarose (Thermo Scientific, Rockford, IL) according to the manufacturer’s instructions (modified to use 0.1 M sodium acetate pH 5.0 for elution of IgG3). Purified mAbs were filter-sterilized, their concentrations were determined by optical density (OD) at 280 nm (1.4 OD_280_ = 1 mg/ml IgG), and their purity was verified by SDS-PAGE. The antigen specificity of Ft mAbs was tested by Western blot as previously described [91] on lysates of Ft LVS or of *E. coli* expressing SchuS4 recombinant GroEL.

### Cloning and Expression of FtGroEL

The nucleotide sequences of SchuS4 GroEL (GenBank accession number 240248234) http://www.ncbi.nlm.nih.gov/genbank/and LVS GroEL (GenBank accession number 89143280) were used for primer design and sequence analysis. A 1,660-bp region was amplified from the genomic DNA of heat-killed LVS or SchuS4 with forward 5′-ATTACATATGGCTGCTAAACAAGTCTTATTTTCAG-3′ and reverse 5′-ACACTCGAGAGACTATTACATCATCCCAGGCATACCGC-3′ primers containing the NdeI and XhoI restriction enzyme cleavage sites (underlined), respectively. The NdeI/XhoI-cleaved PCR product was initially cloned into a PCR 2.1-TOPO vector (Life Technologies, Grand Island, NY) and then transferred into the pET14b T7 expression vector (Novagen, Madison, WI) to add DNA encoding an N-terminal sequence of six histidine residues (a His tag) to the GroEL gene. pET14b (empty vector) and pET14b containing the SchuS4 GroEL gene insert or the LVS GroEL gene insert were separately transformed into One Shot BL21(DE3)pLysS chemically competent *E. coli* (Life Technologies), and induction of protein expression was carried out in LB broth for 4 hours at 37°C in the presence of IPTG (isopropyl-β-d-thiogalactopyranoside) according to manufacturer’s instructions. The bacteria were pelleted and then lysed with BugBuster Master Mix (Novagen) at 10 OD_600_/ml. The lysate was centrifuged at 21,000×g for 10 min at 4°C to remove insoluble components. The generation and expression of FtGroEL mutants are described in File S3.

### ELISA and Western Blot

Direct ELISA and Western blot analysis were performed as previously described [91]. For ELISA on SchuS4, LVS, or TG1, EIA/RIA plates were coated with 100 µl per well of NP40 lysate diluted in 50 mM sodium carbonate buffer pH 9.6 to a final concentration of 0.105 OD_600_ (calculated based on the OD_600_ of the pre-lysis bacterial suspension), which was allowed to dry overnight at 37°C. For ELISA on BL21(DE3)/pLysS that had been transformed with pET14b vector encoding Ft SchuS4 GroEL, or LVS GroEL, or no insert (empty vector), plates were coated with 100 µl per well of BugBuster lysates diluted in 50 mM sodium carbonate buffer pH 9.6 to a final concentration of 0.01 OD_600_ (calculated based on the OD_600_ of the pre-lysis bacterial culture), overnight at 4°C. For ELISA on Hsp60, plates were coated with 100 µl per well of 1 µg/ml of human or mouse recombinant Hsp60 with His tag (Abcam) in 50 mM sodium carbonate buffer pH 9.6, overnight at 4°C. Horseradish peroxidase (HRP)-conjugated anti-mouse IgG1, IgG2a, or IgG3 (heavy chain specific) secondary antibodies (SouthernBiotech, Birmingham, AL) were used as secondary antibodies.

For competition ELISA, plates were coated with 100 µl per well of various dilutions of a GroEL-enriched LVS antigen stock in 50 mM sodium carbonate buffer pH 9.6, overnight at 4°C. The protein concentration of the antigen stock was 0.453 mg/ml as determined by the Bio-Rad DC protein assay (Bio-Rad Laboratories, Hercules, CA). The antigen stock was diluted 1∶500 when Ab53, Ab64 or N200 were used as reporters; 1∶1,000 when N3, N54 or N71 were used as reporters; and 1∶2,000 when Ab12 or N30 were used as reporters. After blocking, plates were incubated for 1 hour with serial dilutions of anti-FtGroEL mAbs before addition of a fixed concentration of reporter mAb – either Ab12 (IgG3, 0.2 µg/ml) for isotype-specific competition ELISA, or the following biotinylated mAbs: Ab53 (1∶8,000 diluted), N3 (1∶4,000 diluted), Ab64 (1∶2,000 diluted), N54 (1∶6,000 diluted), N200 (1∶2,500 diluted), N30 (1∶9,000 diluted), or N71 (1∶3,500 diluted). Biotinylation of mAbs was performed with Lightning-Link Biotin conjugation kit Type A (Innova Biosciences, www.innovabiosciences.com), using 20 µg of antibody per 24 µl reaction volume as per manufacturer’s recommendation. Optimal coating and reporter concentrations (lowest concentrations that yield a>1 OD reading except in the case of Ab64 and N71 that yield a>0.5 OD reading in the ELISA) were pre-determined by testing serial dilutions of reporter on serial dilutions of coating antigen. The ELISA was developed with HRP-conjugated goat anti-mouse IgG3 (γ_3_ chain specific) secondary antibody (SouthernBiotech) or Streptavidin-Peroxidase Polymer (Sigma) for Ab12 or biotinylated mAb reporters, respectively. The binding of each reporter in the presence of graded concentrations of competitor mAbs was determined by OD_450_ measurement. Percent inhibition was determined (after subtracting the blank from all values) using the following formula: [(OD without competitor – OD with competitor)/(OD without competitor)]×100.

For Western blots, precast preparative 4–15% polyacrylamide gradient gels (Bio-Rad Laboratories, Hercules, CA) were loaded with the equivalent of 0.25 OD_600_ of bacterial lysate or with 2.2 µg of human or mouse recombinant Hsp60, and electrophoresis was carried out under denaturing conditions. After transfer to nitrocellulose and treatment of strips with mAbs, the assays were developed with alkaline phosphatase (AP)-conjugated anti-mouse-IgG (H+L) from Promega (Madison, WI) and Western Blue Stabilized Substrate for Alkaline Phosphatase (Promega).

For competition Western blot, nitrocellulose membrane strips were pretreated with 800 µg/ml of Ab12, and then 5 µg/ml of Ab12, Ab53 or Ab64 (primary mAb) was incubated with the membrane strips. AP-conjugates of anti-mouse-IgG (H+L), or of an anti-mouse IgG2a (γ_2a_ specific) (SouthernBiotech) were used as secondary antibodies, and Western Blue Stabilized Substrate for Alkaline Phosphatase was used to visualize binding.

### Antigen Identification by Mass Spectrometry

The target antigen of the Ab64 mAb was identified by immunoprecipitation and mass spectrometric analysis of tryptic peptides derived from the gel-isolated antigen, as follows: An NP40 lysate of LVS was mixed with Ab64 or Ab58 (IgG1, anti-histone-like protein HU form B), or FB11 (anti-FtLPS, IgG2a), followed by capture of Ab64 and any Ab64-bound antigen on Protein G Agarose. After washing and elution with SDS sample buffer, half the sample was subjected to SDS-PAGE under reducing conditions and the other half was subjected to SDS-PAGE under non-reducing conditions, and eluted proteins were stained with SimplyBlue. The gel piece containing the (only) stained band not present in the no-LVS control lane (at 59 kDa apparent molecular weight) from the non-reducing gel was processed by Agnes Bergerat at the Proteomics Core Facility of Boston University School of Medicine (Martin Steffen, Director) for protein(s) identification. The band was excised and subjected to in-gel trypsin digestion. After extraction of peptides from the gel and liquid chromatography on a C18 reverse-phase column, eluted peptides were analyzed by mass spectrometry. The MS/MS spectra were analyzed using SEQUEST software, which identified 33 of 33 peptides as FtGroEL.

### Nucleotide and Deduced Amino Acid Sequence Determination

V region nucleotide sequences of the Ab12 and Ab53 mAbs and of the H chain of the Ab64 mAb were obtained from RT-PCR products generated from the hybridoma cell lines as previously described [93]. V region nucleotide sequences of the other FtGroEL mAbs and of Ab64 L chain were obtained as follows: Primers were modified from Sharon J. et al. [94] to remove restriction enzyme sites and increase internal stability while maintaining optimal sensitivity (modified primers indicated with an “a” suffix). Two new forward primers (VL3 and VL4a) were added to target additional L chain germline genes (see Figure S3 in File S4). RNA was extracted from cultured hybridoma cells as described [93], and cDNA was generated by single-step RT-PCR following manufacturer’s (Qiagen, Limburg, Netherlands) instructions and RT primers (Figure S3 in File S4). Four PCRs were performed for H chain with primer pairs VH1a/CH-γ-LSa, and four for L chain with primer pairs VL1a/CL-κ-LSa using 2 ul of cDNA and 2.5 units of hot-start DNA polymerase (Qiagen) as previously described [95], [96]. Annealing temperatures were varied from 52°C to 57°C during thermal cycling. Amplified cDNAs were sequenced (Genewiz, South Plainfield, NJ) in both sense and antisense with the primer combination that gave the best PCR product. Germline genes (IgBlast, NCBI) with the closest homology to the determined sequences were identified (Table 1).

Homology to immunoglobulin (Ig) germline genes was determined by IgBlast (http://www.ncbi.nlm.nih.gov/igblast/), and conversion to amino acid sequences was done by EMBOSS Transeq (http://www.ebi.ac.uk/Tools/emboss/transeq/index.html).

### *In vivo* Efficacy Studies

All animal procedures were approved by the Boston University Medical Campus Institutional Animal Care and Use Committee. BALB/cJ female mice were obtained from Jackson Laboratories (Bar Harbor, Maine), at 7–8 weeks of age, and inoculated intranasally (i.n.) with Ft bacteria under ketamine/xylazine anesthesia as previously described [32]. For inoculation of mice, Ft bacteria were serially diluted in PBS to the intended CFU/ml based on OD_600_ of the starting stock, and administered in 10 µl followed by 10 µl of PBS as described by Klimpel et al. [97] The actual CFU inoculated per mouse was determined retrospectively after each experiment by plating serial dilutions of the bacterial preparation used for infection on chocolate agar plates. Mice were injected intraperitoneally (i.p.) or injected i.p. and inoculated i.n. with mAb or PBS at specific times before or after SchuS4 infection.

For determination of blood bacterial burden, blood was collected from the submandibular vein into a BD Microtainer tube with Lithium Heparin additive (BD, Franklin Lakes, NJ) 3 days post SchuS4 infection. Undiluted blood and a 5-fold serial dilution of the blood were plated on chocolate agar, and the plates were incubated at 35°C for 2 days for CFU enumeration. Percent CFU reduction compared with PBS was calculated from the median CFU numbers, obtained from the diluted-blood plate if ≥30 or from the undiluted-blood plate otherwise, and the *P* value was determined using the two-tailed Mann Whitney test. *P* values of less than 0.05 were considered statistically significant.

For determination of spleen and lung bacterial burden, mice were euthanized by cervical dislocation (after collection of blood) and spleens and lungs were removed and weighed. Half of the spleen (cut horizontally) or the right part of the lung was further cut into 6 pieces and one piece of spleen or lung was placed in a pre-weighed 1.5-ml Eppendorf Flex-Tubes tube (USA Scientific, Inc., Orlando, FL) containing 50 µl of PBS. The tube was weighed again to obtain the wet tissue weight. Then a pestile for 1.5-ml microcentrifuge tubes (USA Scientific) was used to grind the tissue thoroughly. Two hundred µl of 10,000-fold and 100,000-fold diluted tissue homogenates were plated on chocolate agar plates for CFU determination. After 2-day incubation at 35°C, the 100,000-fold dilution plate was used if it contained ≥30 colonies; otherwise, the 10,000-fold dilution plate was used. Based on the weight of the tissue, CFU/mg wet tissue was calculated and CFU per organ was further deduced using the pre-determined whole-organ weight. Percent CFU reduction compared with PBS was calculated from the median CFU numbers, and the *P* value was determined using the two-tailed Mann Whitney test. *P* values of less than 0.05 were considered statistically significant.

### Generation of FtGroEL, N3, and N200 Homology Models

The FtGroEL homology model was made using the SWISS-MODEL server [98]–[100] and the Deep-View PDB viewer [101]. N3 and N200 homology models were generated using the PIGS server [102]. Before use in docking calculations, the models were minimized using the Protein Preparation Wizard in the Schrodinger suite of programs (Schrodinger, New York, NY).

### Epitope mapping by DXMS

DXMS was performed as previously described [103]. For epitope mapping, two on-exchange sets were prepared: FtGroEL and FtGroEL-antibody (or Fab). Three on-exchange time points were chosen: 10 seconds, 100 seconds and 1000 seconds.

### X-Ray Crystallography

The Fab fragments of Ab53 and Ab64 were made by cleavage of purified IgG using the Pierce Fab Preparation Kit (Thermo, Rockford, IL). The Fabs were concentrated to 27–30 mg/ml in a buffer of 20 mM Tris pH 7.5, 0.15 M NaCl, and 0.02% NaN_3_. Ab53 crystals were grown in sitting drops using the Index Screen HT (Hampton Research, Aliso Viejo, CA) reagent A4 (2 M ammonium sulfate, 0.1 M bistris, pH 6.5) as the precipitant. Ab64 crystals were grown in hanging drops using 1.8 M ammonium sulfate and 0.1 M tris-HCl, pH 8.5 as the precipitant. Prior to freezing in a cold nitrogen stream, the crystals were briefly soaked in a solution of 8∶3 precipitant: cryoprotectant solution (10 M lithium chloride for Ab53 and 85% v/v glycerol for Ab64). X-ray diffraction data were collected on a RAXIS-IV image plate with a Rigaku RU-300 rotating anode as the source of x-rays. Data indexing and processing were performed using the programs DENZO and Scalepack [104] (Table 2) Both crystals contain one Fab molecule in the asymmetric unit. The structures were solved using molecular replacement as implemented in Phenix [105]. The search models were the heavy (H) chain of the catalytic antibody 33F12 (PDB code 1AXT [106]) and the light (L) chain of the anti-HIV Tat protein antibody 11H6H1 (PDB code 3O6K [107]) for Ab53, and the H chain of an antibody specific for a T-cell antigen receptor (PDB code 1KB5 (3)) and the L chain of the Jel42 Fab fragment (PDB code 2JEL (4)) for Ab64. The molecular replacement solutions were then subjected to rebuilding in AutoBuild in Phenix, followed by iterative cycles of refinement and manual rebuilding using the programs Coot [108] and Phenix. The final structures show good geometry and agreement to the diffraction data (Table 2).

### Computational Docking

Protein-protein docking calculations were carried out using the ClusPro 2.0 [109]–[112]. During docking, attractive terms were added to the residues implicated by DXMS as epitope residues, while repulsive terms were added to residues determined to be in the monomer-monomer interface in the tetradecamer, so that poses that are incompatible with the oligomeric state of GroEL were eliminated from consideration.

## Supporting Information

File S1 **Peptide Phage Display Library Screening with Ab53 and Ab64.** **Table S1.** Peptide sequences selected by Ab53 and Ab64 in peptide phage display library screening.
(DOC)

File S2 **Peptide - Antigen Surface Matching and Choice of Mutations to Validate Predicted Ab53 and Ab64 Epitopes. Figure S1.** Binding-site predictions and mutagenesis design.
(DOC)

File S3 **Generation and Expression of FtGroEL Mutants.** **Figure S2A.** PCR primers for generation of FtGroEL point mutants. **Figure S2B.** Western blot (WB) analysis shows abrogation of Ab64 binding by the K344E and Y476E mutations.
(DOC)

File S4 **Supporting figures. Figure S3.** Primers used for Determination of VH and VL Region Nucleotide Sequences. **Figure S4.** DXMS-mapping of FtGroEL Epitopes Targeted by mAbs. Heat-maps and difference heat-maps for determination of FtGroEL DXMS-epitopes targeted by mAbs.
(DOC)
