# Supplementary material for: B-Cell Epitopes in GroEL of Francisella tularensis
Source: PLoS One. 2014 Jun 26;9(6):e99847. doi: 10.1371/journal.pone.0099847 (PMC4072690; doi:10.1371/journal.pone.0099847)
Supplement: File S1 — Peptide Phage Display Library Screening with Ab53 and Ab64. Table S1. Peptide sequences selected by Ab53 and Ab64 in peptide phage display library screening. (DOC) [file pone.0099847.s001.doc]

**File S1.** Peptide Phage Display Library Screening with Ab53 and Ab64

Biopanning was performed as previously described using two linear (7-mer and 12-mer) and a cyclic (7-mer with constrained ends joined by two cysteine residues) phage peptide libraries. Three rounds of biopanning and an immunoblot step were performed, using either Ab53 or Ab64 as the target antibody and mouse polyclonal IgG as negative selection antibody to deplete phage that bind to common parts (constant regions) of the IgG molecule. After the third round of selection and amplification, the log phase *E. coli* were infected with the phage pool to discern individual phage clones. In duplicates, the replica of individual phage colonies was obtained by overlaying nitrocellulose membranes. One membrane was then immunostained with the mAb used as a target for phage panning and the second membrane was stained with a non-specific mAb or mouse polyclonal IgG. The detecting reagents included horseradish peroxidase (HRP)-conjugated goat-anti-mouse IgG followed by chemiluminescent (ECL) substrate (Amersham).

Phage clones that displayed strong signals to the target mAb were grown and further tested by phage ELISA . Briefly, the microtiter plates were first coated with anti-mouse IgG and then either target mAb or irrelevant mAb or mouse polyclonal IgG were captured. Individual phage clones were added to the wells and the bound phage clones were detected by adding HRP-labeled rabbit anti-Fd antibody, followed by addition of an HRP colorimetric enzyme substrate, 2,2-azino-di-[3-ethyl-benzothiaoline sulfonate] (ABTS) to the microtiter wells. Color development in the microtiter wells was measured at 405 nm. Phage clones that gave ELISA signals at least three times the background value were considered positive and their DNAs were subjected to nucleotide sequencing. The predominant translated consensus amino acid sequences that emerged and their frequency are shown in Table S1.

**Table S1** Peptide sequences selected by Ab53 and Ab64 in peptide phage display library screening

| **Peptide Name** | **Sequence** | **Number of clones** |
| --- | --- | --- |
| **Ab53** |  |  |
| 53-7-4 | SYWQKTPHG | 4 |
| 53-7-20 | SYWQKSPTG | 1 |
| 53-c7-3 | CPNKSPLVC | 3 |
| 53-c7-4 | CPNKSPTRC | 1 |
| 53-c7-5 | CPNKSPRIC | 4 |
| 53-12-5 | SSWQSDQHREHMIG | 17 |
| **Ab64** |  |  |
| 64-12-3 | SVPYHDAERFNDG | 2 |
| 64-12-4 | SVPYHDAERFNMDG | 6 |
| 64-12-10 | SLRFDELNFRSVDG | 4 |
| 64-12-15 | SLPEPLSEYDWRRG | 12 |
| 64-7-31 | SFEPWYPRG | 31 |

**References**

1. Sompuram S, Kodela V, Ramanathan H, Wescott C, Radcliffe G, et al. (2002) Synthetic peptides identified from phage-displayed combinatorial libraries as immunodiagnostic assay surrogate quality control targets. Clin Chem 48: 410-420.

2. Sompuram S, Kodela V, Zhang K, Ramanathan H, Radcliffe G, et al. (2002) A novel quality control slide for quantitative immunohistochemistry testing. J Histochem Cytochem 50: 1425-1434.

3. Sompuram S, Vani K, Hafer L, Bogen S (2006) Antibodies immunoreactive with Formalin-Fixed tissue antigens recognize linear protein epitopes. Am J Clin Pathol 125: 82-90.

4. Sompuram S, Vani K, Messana E, Bogen S (2004) A molecular mechanism of formalin fixation and antigen retrieval. Amer J Clin Pathol 121: 190-199.

5. Vani K, Bogen SA, Sompuram SR (2006) A high throughput combinatorial library technique for identifying formalin-sensitive epitopes. J Immunol Methods 317: 80-89.
