# Supplementary material for: B-Cell Epitopes in GroEL of Francisella tularensis
Source: PLoS One. 2014 Jun 26;9(6):e99847. doi: 10.1371/journal.pone.0099847 (PMC4072690; doi:10.1371/journal.pone.0099847)
Supplement: File S2 — Peptide - Antigen Surface Matching and Choice of Mutations to Validate Predicted Ab53 and Ab64 Epitopes. Figure S1. Binding-site predictions and mutagenesis design. (DOC) [file pone.0099847.s002.doc]

**File S2.** Peptide - Antigen Surface Matching and Choice of Mutations to Validate Predicted Ab53 and Ab64 Epitopes

Mapping of the peptide sequences to the surface of the FtGroEL model was carried out using two programs, Pep-3D-Search and the PepSurf server at <http://pepitope.tau.ac.il/index.html> , which use different search algorithms. Both programs start by determination of surface-exposed residues in the protein using the program Surface Racer 5.0 . Each program then searches these exposed residues to determine the path of spatially close residues along the surface of the protein that best matches the input peptides in sequence. Pep-3D-Search utilizes the Ant Colony Optimization algorithm while PepSurf uses a graph alignment approach. These programs consider only a single protein chain for path determination. Since the FtGroEL likely exists as a multimeric complex, residues that were determined to be buried in an interchain interface by manual inspection were removed from consideration. Additionally, FtGroEL may exist in one or both of two different conformations in the complex, so each conformation was run separately through the epitope determination algorithms. The predicted epitopes were: 341-355 for Ab53; and 130-134, 421-428 and 470-489 for Ab64. The Ab64 epitope was mapped regardless of GroEL conformer used; however, Ab53 gave divergent results depending on whether the model was non-GroES-bound or GroES-bound.

The validity of these predicted epitopes was tested by mutational analysis of Ft GroEL. To identify substitutions unlikely to affect overall folding, a list of amino acid preferences at each position in GroEL was created by BLAST alignment (<http://blast.ncbi.nlm.nih.gov/Blast.cgi>) of the 100 GroEL sequences most similar to FtGroEL. This resulted in a table of tolerated, naturally occurring amino acids for each position in the protein. The residues of the predicted Ab53 and Ab64 epitopes were then inspected in the homology model of FtGroEL to determine if these naturally occurring substitutions are likely to affect Ab53 or Ab64 binding. For design of mutants, only the non-GroES bound conformation was considered. Four such naturally occurring mutations were found for Ab64 – A471E, A471K, Y476E and Y476D – and two were found for Ab53 – A343E and K344E. In addition, four other mutations were introduced for Ab64 – three charge reversals and a substitution of leucine by tryptophan. All mutations are shown in Figure S1, the natural mutations on a blue background and the other mutations on a black background.

**Figure S1.** **Binding-site predictions and mutagenesis design.** Putative binding-sites of **Ab53 (red)** and **Ab64 (green)** in Ft GroEL as predicted by peptide – antigen surface matching, and **“natural” mutations (blue)** and **other mutations (black)**.

**References**

1. Huang YX, Bao YL, Guo SY, Wang Y, Zhou CG, et al. (2008) Pep-3D-Search: a method for B-cell epitope prediction based on mimotope analysis. BMC Bioinformatics 9: 538.

2. Mayrose I, Shlomi T, Rubinstein ND, Gershoni JM, Ruppin E, et al. (2007) Epitope mapping using combinatorial phage-display libraries: a graph-based algorithm. Nucleic Acids Res 35: 69-78.

3. Tsodikov OV, Record MT, Jr., Sergeev YV (2002) Novel computer program for fast exact calculation of accessible and molecular surface areas and average surface curvature. J Comput Chem 23: 600-609.
