# Supplementary material for: B-Cell Epitopes in GroEL of Francisella tularensis
Source: PLoS One. 2014 Jun 26;9(6):e99847. doi: 10.1371/journal.pone.0099847 (PMC4072690; doi:10.1371/journal.pone.0099847)
Supplement: File S3 — Generation and Expression of FtGroEL Mutants. Figure S2A. PCR primers for generation of FtGroEL point mutants. Figure S2B. Western blot (WB) analysis shows abrogation of Ab64 binding by the K344E and Y476E mutations. (DOC) [file pone.0099847.s003.doc]

**File S3.** Generation and Expression of FtGroEL Mutants

Point mutants of SchuS4 FtGroEL were generated by overlap PCR , with reverse (R) and forward (F) primers with overlapping ends encoding each mutation, and forward and reverse primers for full-length FtGroEL (1638 nucleotides, starting at methionine) (Figure S2A). The mutated genes were cloned into Topo2.1 and selected clones sequenced, before expression in BL21 *E. coli*, as described for the wild-type gene in Materials and Methods. Lysates of *E. coli* that had been transformed with plasmids encoding wild-type or each of the mutant FtGroEL were tested for FtGroEL expression by SDS-PAGE and for reactivity with the FtGroEL mAbs Ab53, Ab64 or N40 by Western blot analysis (Figure S2B).

**NdeI-GroEL-F** 5’-ATTACATATGGCTGCTAAACAAGTCTTATTTTCAG-3’

**L134W-R** 5’-*gaacacggcttaga****CCA****cgctttta*ACTCTTCAACTAACCTA-3’

**L134W-F** 5’-*taaaagcg****TGG****tctaagccgtgttc*AGATCCAAAATC-3’

**A343E-R** 5’-*attcgttt****TTC****tatcgcttctttttc*ACCAGCACCATC-3’

**A343E-F** 5’-*gaaaaagaagcgata****GAA****aaacga*ATAAATGTAATCAAAGC-3’

**K344E-R** 5’-*attcg****TTC****cgctatcgcttctttttc*ACCAGCACCATC-3’

**K344E-F** 5’-*gaaaaagaagcgatagcg****GAA****cga*a*t*AAATGTAATCAAAGC-3’

**K424D-R** 5’-*taaaccatctagtgc****GTC****ttgtgctcta*AT-3’

**K424D-F** 5’-*tagagcacaa****GAC****gcactagatggttta*ACAGGTG-3’

**K424E-R** 5’-*taaaccatctagtgc****CTC****ttgtgctcta*AT-3’

**K424E-F** 5’-*tagagcacaa****GAG****gcactagatggttta*ACAGGTG-3’

**A471E-R** 5’-*cccttggtt****CTC****tttaacttggttc*ACTACTACAGAAG-3’

**A471E-F** 5’-*gaaccaagttaaa****GAG****aaccaaggg*AACTATGGTTATAATGCTG-3’

**A471K-R** 5’-*cccttggtt****CTT****tttaacttggttc*ACTACTACAGAAG-3’

**A471K-F** 5’-*gaaccaagttaaa****AAG****aaccaaggg*AACTATGGCTATAATGCTG-3’

**Y476E-R** 5’-*gccgcattataccc****TTC****gttcccttg*ATTCGCTTTAACTTGG-3’

**Y476E-F** 5’-*caagggaac****GAA****gggtataatgcggc*AAATGATACTTA-3’

**Y476D-R** 5’-*gccgcattataccc****GTC****gttcccttg*ATTCGCTTTAACTTGG-3’

**Y476D-F** 5’-*caagggaac****GAC****gggtataatgcggc*AAATGATACTTA-3’

**D487R-R** 5’-*ctcaaccat****CCG****cccgtaagtatc*ATTTGC-3’

**D487R-F** 5’-*gatacttacggg****CGG****atggttgag*ATGGG-3’

**XhoI-GroEL-R** 5’-ACACTCGAGAGACTATTACATCATCCCAGGCATACC-3’

**Figure S2A.** **PCR primers for generation of FtGroEL point mutants.** F, forward; R, reverse; restriction enzyme sites NdeI and XhoI underlined; overlapping nucleotides in each primer pair are in lowercase italic; nucleotide replacements are bolded.

**Figure S2B.** **Western blot (WB) analysis shows abrogation of Ab64 binding by the K344E and Y476E mutations.** Lysates of bacteria that had been transformed with vectors encoding wild type (Wild) or the indicated mutants or vector with no insert (Empty) were electrophoresed in four 7.5% SDS polyacrylamide gels (undiluted for SimplyBlue staining and Ab64 WB and 10X diluted for Ab53 and N40 WB). Western blots were reacted with the indicated mAbs and developed with a mixture of anti-mouse-IgG (H+L) + anti-mouse-IgG2a-specific AP conjugates. Note cross-reactivity of Ab53 and N40, but not Ab64, with the EcGroEL (as distinguished in the Empty vector-transformed bacteria).

**References**

1. Madico G, Welsch JA, Lewis LA, McNaughton A, Perlman DH, et al. (2006) The meningococcal vaccine candidate GNA1870 binds the complement regulatory protein factor H and enhances serum resistance. J Immunol 177: 501-510.

2. Madico G, Ngampasutadol J, Gulati S, Vogel U, Rice PA, et al. (2007) Factor H binding and function in sialylated pathogenic neisseriae is influenced by gonococcal, but not meningococcal, porin. J Immunol 178: 4489-4497.
