# Supplementary material for: B-Cell Epitopes in GroEL of Francisella tularensis
Source: PLoS One. 2014 Jun 26;9(6):e99847. doi: 10.1371/journal.pone.0099847 (PMC4072690; doi:10.1371/journal.pone.0099847)
Supplement: File S4 — Supporting figures. Figure S3. Primers used for Determination of VH and VL Region Nucleotide Sequences. Figure S4. DXMS-mapping of FtGroEL Epitopes Targeted by mAbs. Heat-maps and difference heat-maps for determination of FtGroEL DXMS-epitopes targeted by mAbs. (DOC) [file pone.0099847.s004.doc]

**Figure S3.** Primers used for Determination of VH and VL Region Nucleotide Sequences

**Heavy chain**

**VH1a** 5’-GAGGTGCAGCTTCAGGAGTCAGGACCTGGC-3’

**VH2a** 5’-GAGGTCCAGCTTCAGCAGTCTGGGGCTGAGCTTGTGAGGC-3’

**VH3a** 5’-GAAGTGAAACTGCTCGAGTCTGGGGGAGGCTT-3’

**CH-γ-LSa** 5’-TGGGAAGGTATGAACACTGCTGGACAGGG-3’

**CH-γ-RTa** 5’-CTTTGGAGGGAAGATGAAGACGGATGGTC-3’

**Light chain**

**VL1a** 5’-GATGACCCAGTCTCCTTCCTCCCTGTCTGC-3’

**VL2a** 5’-GTTCTCACCCAGTCTCCAGCAATAATGTCAGCATCTC-3’

**VL3** 5’-ATGACCCAGACTCCATCCTCTTTGTCGG-3’

**VL4a** 5’-ATGACCCAAACTCCACTCTCCC-3’

**CL-κ-LSa** 5’-AGAAGCACACGACTGACGCACCTCCAGATG-3’

**CL-κ-RTa** 5’-CATTGATGTCTTTGGGGTAGAAGTTGTTCA-3’

**Figure S4.** DXMS-mapping of FtGroEL Epitopes Targeted by mAbs

**Heat-maps and difference heat-maps for determination of FtGroEL DXMS-epitopes targeted by mAbs.** For a given heat-map, the first row shows residue number, the second row shows residue name and the rest of the rows show protein dynamic features at different time points. As indicated in the color bar, cold colors suggest relatively stable regions, and warm colors suggest relatively flexible regions. All proline residues are shown in white because proline lacks amide hydrogen. Residues not covered by peptides are also shown in white. It should be noted that for a given peptide monitored in the DXMS experiment, the first residue lacks amide hydrogen, the second residue exchanges very fast and any deuteron on-exchange to the peptide will fall off rapidly. Therefore, the first two residues in each peptide are not counted. This is an accepted convention in the DXMS field.
